# Successive Azide and DNA Functionalization for Reversible and Controlled Association of Single‐Wall Carbon Nanotubes

**DOI:** 10.1002/chem.71205

**Published:** 2026-06-01

**Authors:** Razieh Moosavi, Tharun K. Kotammagari, Pia Damlin, Mikko Salomäki, Mika Lastusaari, Han Li, Tuomas Lönnberg

**Affiliations:** ^1^ Department of Chemistry University of Turku Turku Finland; ^2^ Turku Collegium for Science Medicine and Technology University of Turku Turku Finland; ^3^ Department of Mechanical and Materials Engineering University of Turku Turku Finland

**Keywords:** azides, bundling, carbon nanotubes, diazonium salts, DNA, hybridization

## Abstract

A bifunctional linker for robust and straightforward functionalization of carbon‐based nanomaterials, featuring an azido group at one end and a diazonium group at the other, is synthesized and used to introduce azido groups on the sidewalls of (6,5)‐single‐wall carbon nanotubes. The versatility of the azido groups as reactive handles is demonstrated by further functionalization with 6‐carboxyfluorescein and oligonucleotides through copper‐free strain‐promoted azide—alkyne cycloaddition, ideal for biological applications. UV melting profiles and atomic force microscopy (AFM) images of mixtures of nanotubes functionalized with complementary oligonucleotide strands are consistent with hybridization‐controlled bundling—debundling equilibria.

## Graphical abstract

A newly synthesized bifunctional azide—diazonium linker allows robust one‐step introduction of azido groups to single‐wall carbon nanotubes. Versatility of the azido groups as reactive handles is demonstrated by further derivatization with 6‐carboxyfluorescein and oligonucleotides. Nanotubes functionalized with complementary oligonucleotides undergo reversible hybridization‐controlled bundling and debundling.

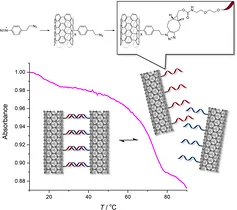

## 1 Introduction

The near‐infrared (NIR) fluorescence of semiconducting single‐wall carbon nanotubes (SWCNTs) makes them an attractive scaffold for biosensing applications as the background interference of biological tissues is minimal in this wavelength range [1, 2]. However, for practical utility, two challenges need to be overcome. First, SWCNT synthesis typically yields a heterogeneous mixture in terms of diameter, chirality, and electronic properties and the sorting of such mixtures is not a trivial task [3, 4, 5, 6, 7, 8]. Second, a robust method for derivatization of SWCNTs with entities allowing specific interaction with a given biomolecular target, such as antibodies or aptamers, is required [1, 9, 10].

The first challenge has been tackled through development of controlled synthesis [7, 11, 12] as well as sophisticated separation methods [6, 13, 14, 15, 16, 17], although large‐scale production of single‐chirality SWCNTs remains elusive. Early attempts at the second one were inspired by the observation that the acidic oxidation in a mixture of sulfuric and nitric acid used in the purification of SWCNTs introduced carboxyl groups not only at the ends but also on the sidewalls [18, 19, 20, 21]. These conditions are rather harsh and actually yield a mixture of carboxyl, carbonyl, phenol, and sulfate functionalities [22]. Treatment with nitrenes, carbenes, azomethine ylides, or radicals is somewhat more controlled and has been used to introduce a number of interesting modifications [23, 24, 25]. However, most of these studies have focused on modifications to solubilize SWCNTs or adjust their electronic properties, rather than reactive handles that would readily lend themselves to further functionalization.

**FIGURE 1:**
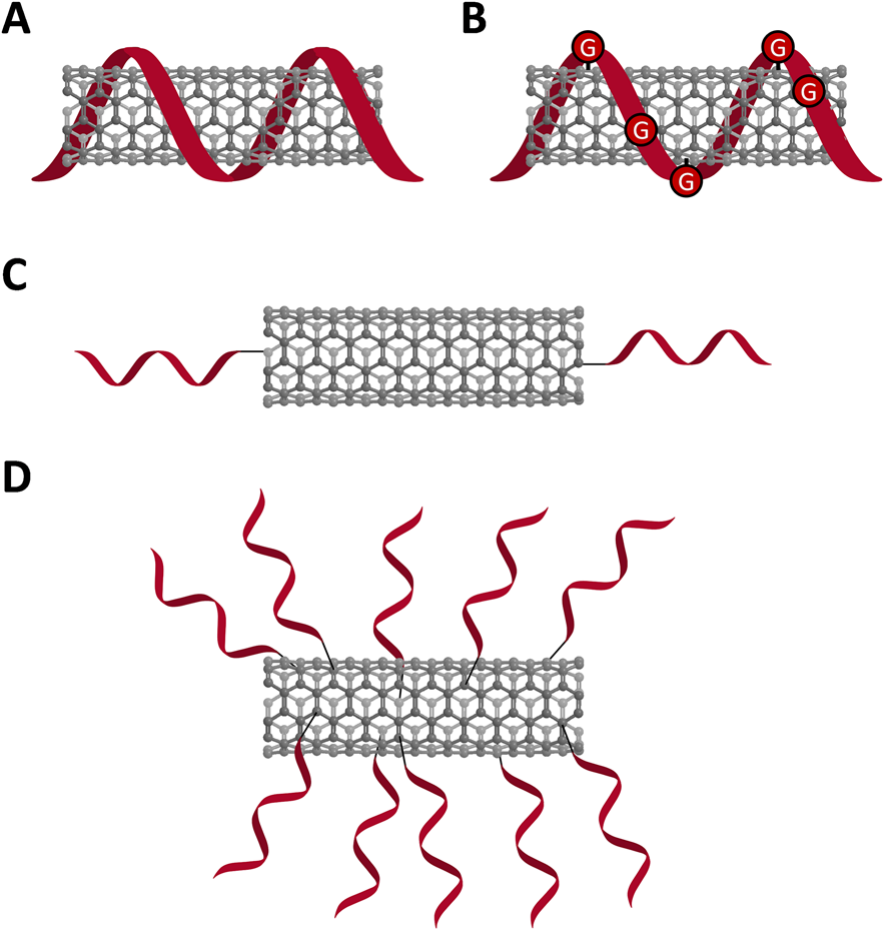
Alternative strategies for functionalizing SWCNTs with DNA: (A) noncovalent wrapping of DNA around a SWCNT [26, 27]; (B) covalent cross‐linking of DNA and a SWCNT through guanine residues [28, 29] and covalent attachment of DNA oligonucleotides at (C) the ends [31] or (D) the sidewalls of SWCNTs.

As our interest lies in functionalization of SWCNTs with oligonucleotides, it is worth casting a closer look at the state‐of‐the art of this particular field. DNA spontaneously wraps itself around SWCNTs in a noncovalent but sequence‐dependent manner, mainly through stacking interactions (Figure 1A) [26, 27]. Exposure of such assemblies to singlet oxygen generates covalent links between guanine bases and the SWCNT (Figure 1B) [28, 29, 30]. This type of DNA functionalization is useful for dispersion and solubilization of SWCNTs but less so for construction of sensors that leverage the programmable recognition properties of DNA. Recently, covalent functionalization of the ends of SWCNTs with DNA oligonucleotides featuring terminal azido groups through formation of aziridine photoadducts has been reported (Figure 1C) [31]. The sequence information of the DNA tags has been successfully harnessed for hybridization‐driven formation of SWCNT junctions [31, 32] as well as SWCNT—protein hybrids [33]. However, for applications requiring a higher oligonucleotide density or closer contact of the SWCNTs with each other or a target molecule, the complementary approach of sidewall functionalization (Figure 1D) would also be in demand. With pre‐carboxylated SWCNTs, this idea has been realized through a three‐step process involving silanation with a bromoalkyltrichlorosilane, displacement of the bromide with an azide and, finally, strain‐promoted azide—alkyne cycloaddition (SPAAC) [34] with cyclooctyne‐functionalized oligonucleotides [35].

Among the approaches reported for derivatization of SWCNTs, aryl diazonium functionalization is widely used and highly influential, creating luminescent sp^3^ quantum defects and enabling applications from photoluminescence enhancement to biosensing [36, 37, 38, 39]. The mechanism is generally described as electron‐transfer–initiated aryl radical formation, followed by radical addition to the SWCNT sidewall. The aryl radical may form either by electrochemical reduction [40] or by catalyst‐free thermal/photochemical initiation (similar to the Gomberg–Bachmann reaction) [41, 42]. For the present study, we chose the latter because of its simplicity and functional group compatibility. Azide was selected as the functional group at the other end of the linker to allow further functionalization with a wide range of compounds by the ubiquitous SPAAC reaction [34]. The versatility of this method was demonstrated by functionalization of (6,5)‐SWCNTs with 6‐carboxyfluorescein (6‐FAM) and two different oligonucleotides. Compared to the previously reported method [35] for azide functionalization of SWCNTs, the method presented herein is simpler, eliminating the intermediary step of conversion of alkyl bromides to alkyl azides. Furthermore, as functionalization with diazonium salts does not rely on prior carboxylation but can be performed on pristine nanotubes, we could fully leverage our expertise in advanced separation techniques [14, 15, 16, 17], as demonstrated by the use of single‐chirality (6,5)‐SWCNT (Figure S1 in the Supporting Information) as the starting material. Finally, UV melting experiments confirmed hybridization‐controlled association of the oligonucleotide‐functionalized (6,5)‐SWCNTs. The ability to control and monitor hybridization on the nanotube surface not only enhances the functional versatility of SWCNTs but also opens avenues for integrating them into next‐generation bioelectronic, optoelectronic, and nanomedical devices.

## 2 Results and Discussion

### 2.1 Synthesis of the Azide–Diazonium Salt Linker

**SCHEME 1:**
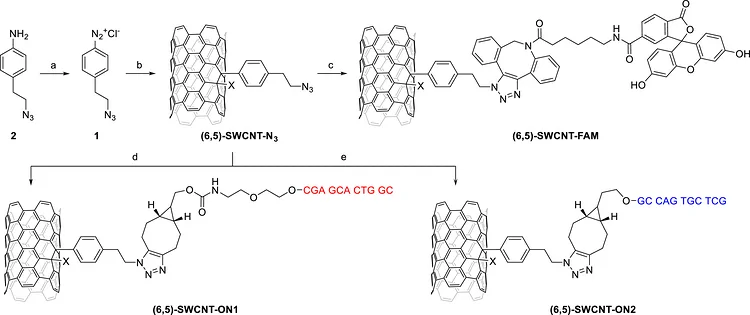
Synthesis of the azide–diazonium salt linker **1** and its application to functionalize (6,5)‐SWCNTs first with azido groups and then with 6‐carboxyfluorescein or oligonucleotides. Reagents and conditions: (a) NaNO_2_, DCl, D_2_O, CD_3_OD, 25°C, 5 min; (b) (6,5)‐SWCNT, SDS, H_2_O, 25°C, 16 h; (c) DBCO‐6‐FAM, H_2_O, 55°C, 3 d; (d) **ON1**, H_2_O, 55°C, 3 d; (e) **ON2**, H_2_O, 55°C, 3 d. The so‐called pairing group “X” remains obscure but likely candidates include OH or another 4‐(2‐azidoethyl)phenyl group [45].

Scheme 1 outlines the synthesis of the bifunctional azide–diazonium salt linker **1** needed for azide derivatization of SWCNTs. Linker **1** represents a minimal structure featuring an aryldiazonium and an alkyl azide function electronically decoupled from each other. Furthermore, synthesis of the aniline starting material **2** has been described in the literature [43]. Diazotation of compound **2** was accomplished by treatment with an equimolar amount of sodium nitrite in the presence of hydrochloric acid. Given the known reactivity of diazonium salts, no attempts were made to isolate compound **1** but the reaction solution was used as such after characterization by nuclear magnetic resonance (NMR, Figures S2, S3 in the Supporting Information) and high‐resolution mass spectrometry (HRMS). The sample was kept at −20°C and analyzed again after three months. At that point, the NMR spectra were essentially identical to those obtained immediately after diazotation.

### 2.2 Azide Functionalization of (6,5)‐SWCNTs

**FIGURE 2:**
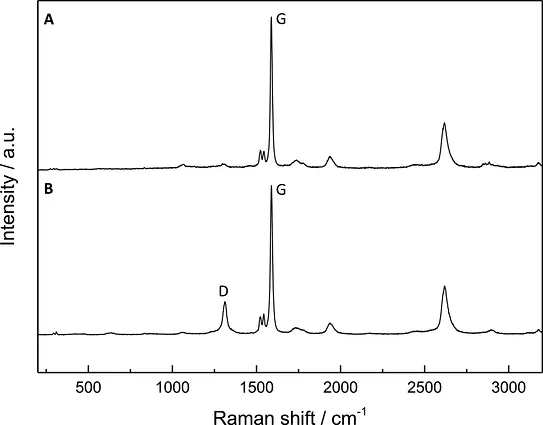
Representative Raman spectra of (6,5)‐SWCNTs (A) before and (B) after treatment with compound **1**. The appearance of a prominent D peak at 1310 cm^−1^ indicates formation of defects.

Aqueous dispersions of (6,5)‐SWCNTs in 1% (*m*/*v*) sodium dodecyl sulfate (SDS) were allowed to react with the azide—diazonium salt linker **1** overnight (Scheme 1). After filtration and thorough washing with water to remove the excess linker, Raman spectroscopic analysis (Figure 2) revealed the emergence of a defect‐induced D band centered at 1310 cm^−1^, indicative of covalent modification of the nanotube sidewalls [44]. After aryl addition to the SWCNT sidewall, the resulting nanotube‐derived radical or cationic intermediate may be stabilized by delocalization over the extended π system and eventually undergo further reaction depending on the medium. Possible termination pathways include reaction with H‐ or OH‐containing species, nucleophilic trapping by solvent, or formation of aryl‐paired defects [45]. A defect density of 150  ± 10 µm^−1^ was determined by comparison of the ratio of the areas of the D and G (at 1590 cm^−1^) peaks in pristine and functionalized (6,5)‐SWCNTs, using the quantification method of Finn et al. [44]. This method provides a semi‐quantitative measure of defect density, although curvature and chirality may influence the absolute values.

### 2.3 Reaction of Azide Functionalized (6,5)‐SWCNTs With DBCO Functionalized 6‐Carboxyfluorescein

**FIGURE 3:**
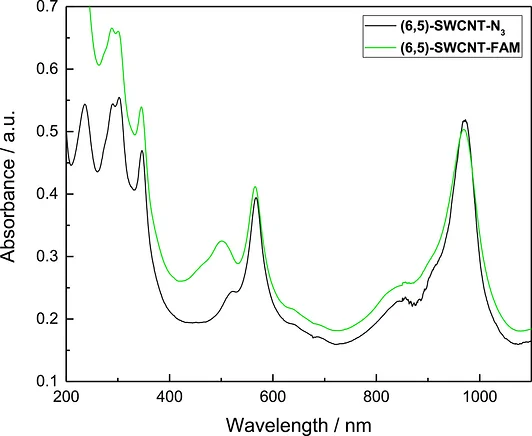
UV‐vis spectra of functionalized nanotubes **(6,5)‐SWCNT‐N_3_** (black line) and **(6,5)‐SWCNT‐FAM** (green line) dispersed in 1% (*m*/*v*) aqueous SDS. The new peak at 501 nm confirms covalent attachment of 6‐carboxyfluorescein.

Further derivatization of the azide functionalized (6,5)‐SWCNTs (**(6,5)‐SWCNT‐N_3_**) through SPAAC was first tested with a commercially available 6‐carboxyfluorescein (6‐FAM) reagent bearing an azadibenzocyclooctyne (DBCO) group (Scheme 1). 6‐FAM was chosen because of its high absorptivity at a wavelength where (6,5)‐SWCNTs are largely transparent (495 nm). To ensure reasonable conversion despite the relatively low azide concentration (estimated as 1.7 µM), the reaction was carried out at elevated temperature (55°C) and allowed to run for 3 days. The nanotubes were recovered by filtration and washed with water to remove the excess DBCO‐6‐FAM reagent. Even after thorough washing, the UV spectrum of the product mixture (Figure 3) showed a clear peak at 501 nm, confirming formation of the desired product **(6,5)‐SWCNT‐FAM** and thus the presence of reactive azido groups on the starting material **(6,5)‐SWCNT‐N_3_**.

### 2.4 Reaction of Azide Functionalized (6,5)‐SWCNTs With BCN Functionalized Oligonucleotides

Encouraged by the successful reaction of **(6,5)‐SWCNT‐N_3_** with DBCO‐6‐FAM, we next set out to derivatize these nanotubes with DNA oligonucleotides (Scheme 1). Two mutually complementary sequences (**ON1**, 5´‐CGAGCACTGGC‐3´ and **ON2**, 5´‐GCCAGTGCTCG‐3´) were employed, each bearing a bicyclo[6.1.0]non‐4‐yne (BCN) group at its 5´‐terminus. In **ON1**, this reactive group was tethered to the oligonucleotide through a carbamate‐linked aminoethoxyethanol spacer, while **ON2** featured a much shorter ethylene linker in the same place. Reaction conditions and isolation of the products **(6,5)‐SWCNT‐ON1** and **(6,5)‐SWCNT‐ON2** were identical to those described above for **(6,5)‐SWCNT‐N_3_**.

**FIGURE 4:**
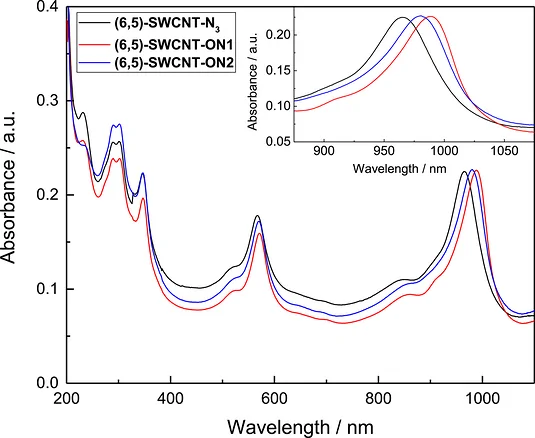
UV‐vis spectra of functionalized nanotubes **(6,5)‐SWCNT‐N_3_** (black line), **(6,5)‐SWCNT‐ON1** (red line) and **(6,5)‐SWCNT‐ON2** (blue line); pH = 7.4 (20 mM cacodylate buffer); *I*(NaCl) = 0.10 M; 1% (*m*/*v*) SDS. The inset shows the red‐shift of the peak at around 1000 nm in more detail.

UV‐vis‐NIR spectra (Figure 4) showed a 15–25 nm red‐shift of the (6,5) S_11_ band (around 1000 nm) after introducing oligonucleotides. This magnitude and direction are consistent with increased local dielectric screening at the nanotube surface as SDS is displaced by hydrated, polyanionic oligonucleotides, which lowers the E_11_ energy [46, 47, 48]. Indeed, no red‐shift of the 1000 nm peak was observed on derivatization of **(6,5)‐SWCNT‐N_3_** with the nonpolar DBCO‐6‐FAM. The broadening of the S_11_ transition (Figures 3 and 4) reflects the ensemble heterogeneity inherent to covalent sp^3^ functionalization at the defect density used here (150 µm^−^^1^, or one defect per 6.7 nm). At this density, distributions of local binding configurations, proximal‐defect clustering, and surface dielectric environments collectively contribute to the observed spectral width, in line with earlier reports on aryl‐functionalized SWCNTs [49, 50, 51]. In contrast to **(6,5)‐SWCNT‐FAM**, direct detection of the oligonucleotides based on their contribution to the UV spectrum was difficult owing to the high absorptivity of the nanotubes in this wavelength range (around 260 nm).

### 2.5 UV Melting Profiles of the Functionalized (6,5)‐SWCNTs

**FIGURE 5:**
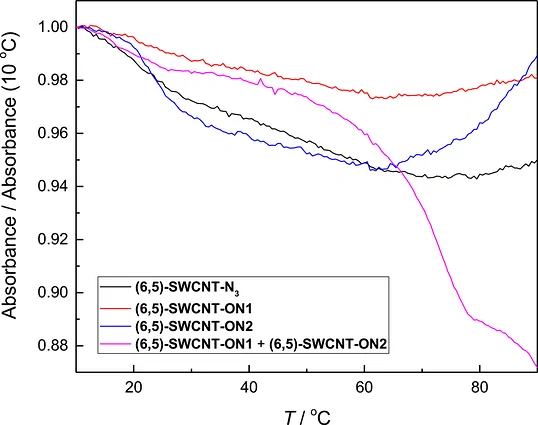
UV melting profiles of **(6,5)‐SWCNT‐N_3_** (black line), **(6,5)‐SWCNT‐ON1** (red line), **(6,5)‐SWCNT‐ON2** (blue line), and an equimolar mixture of **(6,5)‐SWCNT‐ON1** and **(6,5)‐SWCNT‐ON2** (magenta line);  *ρ*(nanotubes) = 550 µg L^−1^; pH = 7.4 (20 mM cacodylate buffer); *I*(NaCl) = 0.10 M; 1% (*m*/*v*) SDS. Each line represents an average of three cooling curves and standard deviation of each data point is less than 1%.

The complementary oligonucleotides of **(6,5)‐SWCNT‐ON1** and **(6,5)‐SWCNT‐ON2** would be expected to hybridize with each other and thus cause bundling of the nanotubes even in the presence of surfactants. Such bundling—and debundling—could be detectable as a change in the UV‐vis spectrum of the nanotube mixture. To test this hypothesis, UV melting profiles (Figure 5) of **(6,5)‐SWCNT‐ON1**, **(6,5)‐SWCNT‐ON2** and their equimolar mixture were recorded at 260 nm over a range of 10°C–90°C. As neither of the two oligonucleotides is self‐complementary, but can only form a double helix with the other one, samples containing only **(6,5)‐SWCNT‐ON1** or **(6,5)‐SWCNT‐ON2** serve as negative controls—nanotubes functionalized with oligonucleotides but with no propensity for hybridization‐driven bundling. A sample of **(6,5)‐SWCNT‐N_3_** was also included as a negative control lacking any oligonucleotides. In each sample, the mass concentration of a single type of functionalized nanotube was approximately 550 µg L^−1^. From this number, the defect density of 150 µm^−1^ and the fact that 1 µm of a (6,5)‐SWCNT contains 88000 carbon atoms, concentration of the oligonucleotide strands can be estimated as 0.08 µM.

Between 10°C and 60°C, the absorbance of all samples decreased. At higher temperatures, the absorbance of **(6,5)‐SWCNT‐N_3_** and **(6,5)‐SWCNT‐ON1** remained nearly constant, while that of **(6,5)‐SWCNT‐ON2** recovered with no sign of saturation below 90°C. The different behavior of **(6,5)‐SWCNT‐ON1** and **(6,5)‐SWCNT‐ON2** at temperatures exceeding 60°C could be tentatively related to the different lengths of the linker between the nanotube sidewall and the oligonucleotide. In particular, the absorbance recovery observed for **(6,5)‐SWCNT‐ON2** could reflect temperature‐induced weakening of non‐covalent interactions, including π‐stacking, between the nucleobases and the nanotube sidewall, possibly accompanied by reorganization of the local SDS shell. Such effects may be more pronounced for **(6,5)‐SWCNT‐ON2** because its shorter linker places the oligonucleotide closer to the nanotube surface. In any case, the sample that really stood out was the mixture of **(6,5)‐SWCNT‐ON1** and **(6,5)‐SWCNT‐ON2**, exhibiting sigmoidal decrease of absorbance, with a melting temperature of approximately 72°C. In other words, the mixture of **(6,5)‐SWCNT‐ON1** and **(6,5)‐SWCNT‐ON2** behaved completely differently from either of these oligonucleotide functionalized nanotubes alone. As other conditions (e.g., surfactant, pH and ionic strength) between the samples were equal, hybridization of the oligonucleotide chains emerges as the most probable reason behind the unique melting profile of the mixture of **(6,5)‐SWCNT‐ON1** and **(6,5)‐SWCNT‐ON2**. Finally, it should be pointed out that while the heating curves were superimposable with each other and the cooling curves with each other over at least three cycles, samples containing oligonucleotide‐functionalized nanotubes (especially **(6,5)‐SWCNT‐ON2**) showed considerable hysteresis between the heating and cooling curves (Figure 5 shows only averaged cooling curves but all curves are presented in Figures S6–S9 of the Supporting Information).

At first glance, the thermal hypochromicity observed with the mixture of **(6,5)‐SWCNT‐ON1** and **(6,5)‐SWCNT‐ON2** may seem counterintuitive as unwinding of a double‐helical oligonucleotide is associated with thermal hyperchromicity at 260 nm. However, one should bear in mind that even at this wavelength, the absorption band is dominated by the SWCNT π‐plasmon and contribution of the oligonucleotides is only minor [52]. Furthermore, bundling of SWCNTs is known to increase the UV baseline, especially at short wavelengths [53, 54]. In a mixture of **(6,5)‐SWCNT‐ON1** and **(6,5)‐SWCNT‐ON2**, the bundling would be driven by hybridization of the complementary oligonucleotides **ON1** and **ON2**. At sufficiently high temperatures, the **ON1**•**ON2** duplexes melt, leading to debundling of the nanotubes and thus the observed sigmoidal decrease in absorbance. Hybridization‐driven bundling of SWCNTs has been reported previously [55] but in that case the DNA strands were attached to the SWCNTs either non‐covalently (Figure 1A) or through guanine cross‐links (Figure 1B) and debundling was achieved through strand‐displacement, rather than thermal denaturation.

The melting temperature of 72°C determined for the mixture of **(6,5)‐SWCNT‐ON1** and **(6,5)‐SWCNT‐ON2** is approximately 20°C higher than what has been reported [56] for duplexes of similar length and sequence under the same conditions. In all likelihood, this difference reflects cooperative hybridization of the oligonucleotide strands: each nanotube is derivatized with several (75 assuming an average length of 500 nm although obviously many would be pointing away from the other nanotube) and debundling requires dissociation of all (or at least most) of the duplexes. The observed hysteresis is also consistent with such a binding model.

### 2.6 Atomic Force Microscopy of the Functionalized (6,5)‐SWCNTs

After the three heating and cooling cycles of the UV melting experiments, visible aggregation of the SWCNTs was observed in all samples (Figure S10 in the Supporting Information). The aggregation was particularly prominent in the sample containing a mixture of **(6,5)‐SWCNT‐ON1** and **(6,5)‐SWCNT‐ON2**, consistent with the hybridization‐driven bundling discussed above. To further elucidate this point, each sample was analyzed by atomic force microscopy (AFM).

**FIGURE 6:**
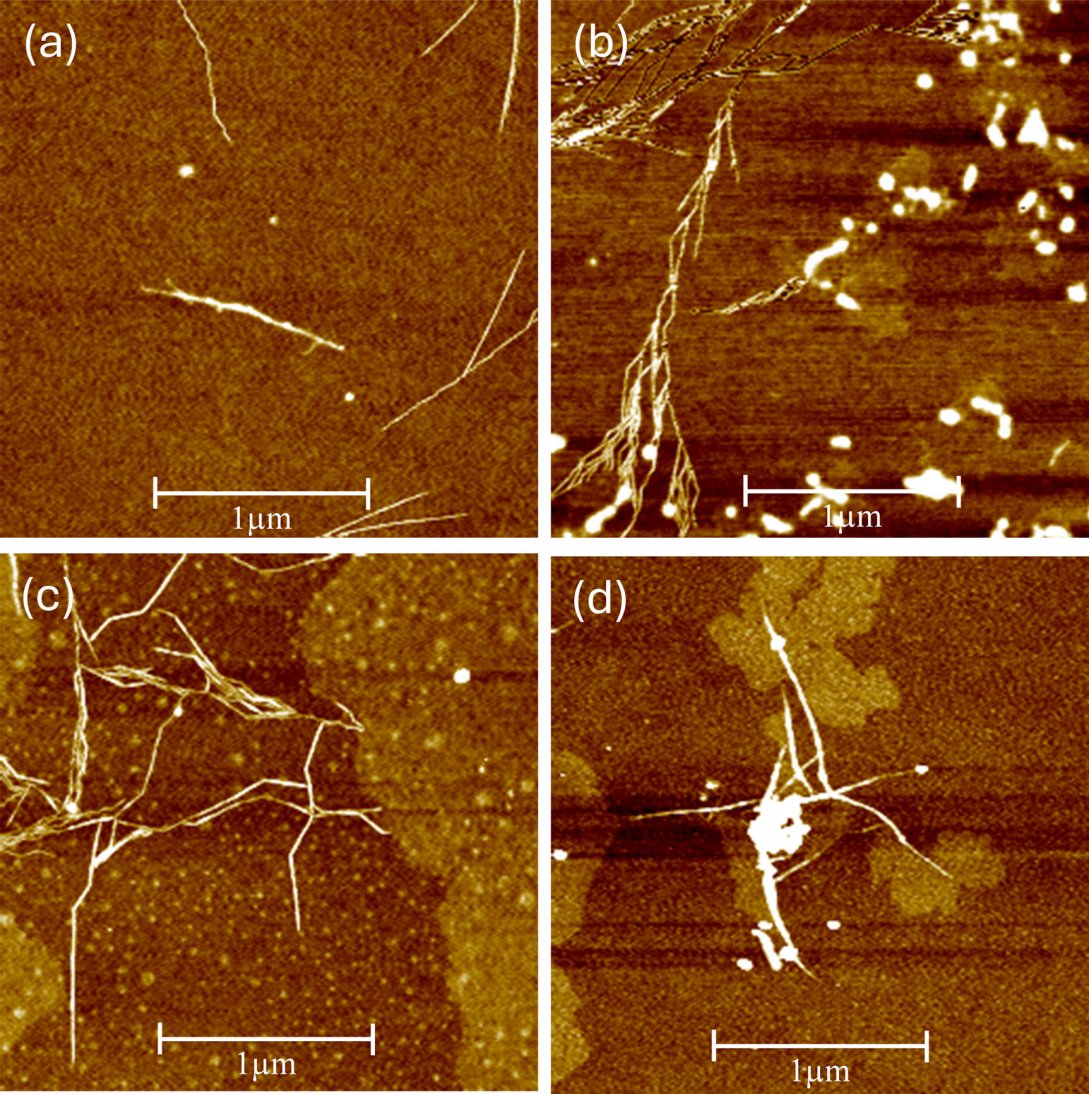
AFM images of (A) **(6,5)‐SWCNT‐N_3_**, (B) **(6,5)‐SWCNT‐ON1**, (C) **(6,5)‐SWCNT‐ON2** and (D) an equimolar mixture of **(6,5)‐SWCNT‐ON1** and **(6,5)‐SWCNT‐ON2**.

AFM images of the sample containing **(6,5)‐SWCNT‐N_3_** showed mainly scattered thin lines (Figure 6A), with a length of approximately 0.5 µm and a height of 0.8 nm (height profile analysis presented in Figures S11–S14 in the Supporting Information). These dimensions are in perfect agreement with the length and diameter of individual (6,5)‐SWCNTs. Some thicker (height of approximately 2 nm) lines were also present, consistent with aggregates of two nanotubes.

Thin lines corresponding to individual nanotubes were the dominant feature also in the samples containing **(6,5)‐SWCNT‐ON1** or **(6,5)‐SWCNT‐ON2**, but many more end‐to‐end or end‐to‐side junctions could be seen (Figure 6B,C). With **(6,5)‐SWCNT‐ON1**, these junctions led to formation of elaborate bifurcated structures, whereas with **(6,5)‐SWCNT‐ON2** the aggregation pattern appeared to be more random. The prevalence of end‐to‐end and end‐to‐side junctions suggests interactions of the terminal functional groups of the nanotubes with each other or with the covalently attached oligonucleotides. The reasons behind the different morphology of the aggregates formed by **(6,5)‐SWCNT‐ON1** and **(6,5)‐SWCNT‐ON2** remain elusive but could be related to the different length of the linker between the nanotube and the oligonucleotides.

AFM images of the sample containing an equimolar mixture of **(6,5)‐SWCNT‐ON1** and **(6,5)‐SWCNT‐ON2** were distinct from those of all the other samples. Firstly, the surface was largely devoid of any structures resembling nanotubes and only a few isolated clusters could be found (Figure 6D). Similar results have been reported previously for mixtures of nanotubes and complementary DNA oligonucleotides and interpreted as hybridization‐driven bundling [55]. Secondly, while the length of the constituent structures within these clusters matched that of (6,5)‐SWCNTs, most of them were much thicker than even the thickest counterparts in the other samples. Heights corresponding to the diameter of individual nanotubes were not observed at all and the thickest structures showed heights of up to 6 nm, consistent with bundles of seven or more. In other words, the AFM analysis corroborates our interpretation of the UV melting profiles in terms of hybridization‐driven bundling, which is only possible with the mixture of **(6,5)‐SWCNT‐ON1** and **(6,5)‐SWCNT‐ON2**.

### 2.7 Photoluminescence Properties of the Functionalized (6,5)‐SWCNTs

Sidewall functionalization through reaction with aryldiazonium salts inevitably introduces defects in the SWCNTs and is thus detrimental to their photophysical properties. To find out to what extent the functionalized (6,5)‐SWCNTs used in the present study retained theirs, steady‐state fluorescence spectra (Figure S16 in the Supporting Information) of **(6,5)‐SWCNT‐N_3_** and the pristine **(6,5)‐SWCNT** starting material were recorded. The spectrum of the pristine starting material showed a strong characteristic E_11_ emission peak at approximately 990 nm. In the spectrum of the azide functionalized product **(6,5)‐SWCNT‐N_3_**, this peak was largely diminished (to approximately 5% intensity) but could nonetheless be detected at the same wavelength.

## 3 Conclusion

We have developed a straightforward and robust technique for covalent functionalization of single‐walled carbon nanotubes (SWCNTs) with azido groups. The method introduces covalent sidewall defects while retaining the characteristic NIR excitonic absorption of the (6,5)‐SWCNTs, which remains useful for monitoring subsequent functionalization and hybridization‐driven assembly. Marked loss of the E_11_ emission is an issue that will be addressed in future studies by exploring lower functionalization (and thus defect) densities. The azido group is an extremely versatile reactive handle as it allows further derivatization through either Cu(I)‐ or strain‐promoted azide—alkyne cycloaddition with an essentially limitless variety of alkyne‐functionalized molecules, many of which are commercially available. As an example, we have demonstrated functionalization of (6,5)‐SWCNTs with 6‐carboxyfluorescein and two different oligonucleotides, each one bearing a different cyclooctyne group. (6,5)‐SWCNTs functionalized with complementary oligonucleotides underwent reversible hybridization‐driven bundling, confirmed by both UV melting and AFM experiments. In addition to the intended application in biosensors, we believe that our method will find use in construction of hybrid nanostructures of DNA and SWCNTs.

## 4 Experimental Section

### 4.1 General Methods

Reagents and solvents were commercial products that were generally used as received. The only exception was triethylamine, which was distilled before being used in the preparation of high‐performance liquid chromatography (HPLC) eluents. NMR spectra were recorded on a Bruker Biospin 500 MHz NMR spectrometer, with chemical shifts referenced to the corresponding residual signal of the solvent. Mass spectra were recorded on a Waters ACQUITY RDa mass spectrometer. For UV‐vis spectrophotometry, several instruments were used, including PerkinElmer Lambda 35, Shimadzu UV‐1900, Jenway Genova Nano and JASCO V‐770 spectrophotometers.

### 4.2 4‐(2‐Azidoethyl)Benzenediazonium Chloride (**1**)

4‐(2‐Azidoethyl)aniline [43] (**2**, 0.100 g, 0.617 mmol) was dissolved in a mixture of D_2_O (500 µL) and CD_3_OD (500 µL). A 35% solution of DCl in D_2_O (134 µL) was added, followed by NaNO_2_ (38.5 mg, 0.558 mmol). Complete conversion of the starting material was verified by NMR spectrometric analysis of the reaction solution, which was used as such in further reactions. ^1^H NMR (500 MHz, D_2_O/CD_3_OD) δ 8.62 (d, *J* = 8.5 Hz, 2H, Ar‐H2 & H6), 7.92 (d, *J* = 8.3 Hz, 2H, Ar‐H3 & H5), 3.74 (t, *J* = 6.4 Hz, 2H, N_3_CH_2_), 3.19 (t, *J* = 6.3 Hz, 2H, ArCH_2_). ^13^C NMR (126 MHz, D_2_O/CD_3_OD) δ 157.2 (Ar‐C4), 133.9 (Ar‐C2 & C6), 133.8 (Ar‐C3 & C5), 113.1 (Ar‐C1), 52.2 (N_3_CH_2_), 37.0 (ArCH_2_). HRMS (ESI^+^‐TOF): *m*/*z* calculated for [C_8_H_8_N_5_]: 174.07742; found: 174.07558 [M‐Cl]^+^.

### 4.3 Separation of (6,5)‐SWCNTs

Single‐chirality (6,5)‐SWCNTs were prepared as described previously [14, 17] by aqueous two‐phase extraction (ATPE). Briefly, SWCNT powder (CoMoCat65i, Sigma–Aldrich) was dispersed at 1 mg mL^−^^1^ in 2% (*m*/*v*; 20 g L^−^^1^) sodium deoxycholate (DOC, Sigma–Aldrich) by tip sonication (45 min, ice bath). After centrifugation (16,639 × g, 1 h, Eppendorf 5810), the upper ∼80% of the supernatant was collected as the parent dispersion. ATPE was then performed following our established protocol [14] using a dextran (70 kDa, TCI)/poly(ethylene glycol) (PEG, 6 kDa, Alfa Aesar) two‐phase system. Sorting employed co‐surfactant systems based on DOC, sodium cholate (SC, Sigma–Aldrich), and sodium dodecyl sulfate (SDS, Sigma–Aldrich). DOC was held at 0.05% (*m*/*v*), while SDS was increased gradually from 1.3 to 1.5% (*m*/*v*) to selectively partition (6,5)‐SWCNTs into the PEG‐rich top phase. To remove residual metallic species, an additional semiconducting/metallic partitioning step was carried out with SC (0.9%), SDS (1.0%), DOC (< 0.02%) and sodium hypochlorite (NaClO, 10–15% available chlorine). Purified (6,5) fractions were finally concentrated and exchanged to 1% (*m*/*v*) SDS by iterative ultrafiltration using a stirred cell (Millipore) with a 300 kDa membrane.

### 4.4 Raman Spectroscopy

Raman measurements were performed using a Renishaw inVia confocal Raman microscope (Leica). A 532 nm laser was employed for excitation, with laser power intensities ranging from 0.1% to 5%, depending on the sample sensitivity. Spectra were acquired using a 50 × objective lens, unless otherwise stated. Raman‐scattered light was dispersed using an 1800 lines/mm diffraction grating and detected with a Peltier‐cooled charge‐coupled device (CCD) detector. Spectra were collected over a range of 150–3200 cm^−1^. The spectrometer was calibrated using a silicon standard, with a reference peak at 520.5 cm^−^^1^. Samples were prepared by drop‐casting 20 µL of SWCNT solution mixed with 20 µL of isopropanol onto cleaned glass slides. The mixture promoted aggregation, facilitating easier identification under the Raman microscope.

### 4.5 Azide Functionalization of (6,5)‐SWCNTs

Purified (6,5)‐SWCNTs were suspended in a 1% (*m*/*v*) aqueous solution of SDS to give 150 mL of a dispersion with an absorbance of approximately 1 for the S_11_ transition (around 1000 nm). A solution of compound **1** in a mixture of D_2_O and CD_3_OD (3.0 µL, 1.6 µmol) was added and the resulting mixture stirred for 16 h at 25°C, protected from light. The dispersion was then filtered and washed by stirred‐cell ultrafiltration (Amicon, Merck Millipore; 300 kDa MWCO membrane) to remove excess compound **1**. The **(6,5)‐SWCNT‐N_3_** recovered by filtration was resuspended in a 1% (*m*/*v*) aqueous solution of SDS to give 15 mL of a dispersion with an absorbance of approximately 10 for the S_11_ transition (at 971 nm). Raman spectra of the product showed a prominent peak at 1310 cm^−1^, confirming defect formation. Defect density was calculated using equation (1) [44].

(1) $$n_{d}=414\;\mu m^{-1}\left(\frac{I_{D,\;\mathrm{product}}}{I_{G,\mathrm{product}}}-\frac{I_{D,\mathrm{staring}\;\mathrm{material}}}{I_{G,\mathrm{starting}\;\mathrm{material}}}\right)$$

*I*_D_ and *I*_G_ are integrals of the D (1310 cm^−1^) and G (1590 cm^−1^) peaks in the Raman spectra of the product and the starting material. Averages of five spectra were used in the calculation.

A second, smaller, batch (approximately 6% of the batch discussed above) of **(6,5)‐SWCNT‐N_3_** was prepared under the same conditions to test the reproducibility of the functionalization process. Representative Raman spectra of both batches are presented in Figure S15 of the Supporting Information. Defect densities of the large and the small batch were approximately 150 and 110 µm^−1^, indicating moderate batch‐to‐batch variation in the extent of functionalization. Accordingly, the defect density of each batch should be checked before subsequent SPAAC functionalization, particularly when quantitative comparison between batches is required.

### 4.6 Reaction of Azide Functionalized (6,5)‐SWCNTs With DBCO‐6‐FAM

20 mM solution of the DBCO‐6‐FAM reagent in *N*,*N*‐dimethylformamide (DMF) (0.42 µL, 8.4 nmol) was added to a dispersion of **(6,5)‐SWCNT‐N_3_** (*A* = 10 at S_11_) in 1% (*m*/*v*) aqueous SDS (1.0 mL). The mixture was incubated protected from light at 55°C for 3 d, after which it was purified by stirred‐cell ultrafiltration to remove unreacted dye. The **(6,5)‐SWCNT‐FAM** recovered by filtration was resuspended in a 1% (*m*/*v*) aqueous solution of SDS and the resulting dispersion analyzed by UV‐vis‐NIR spectrophotometry. Appearance of a prominent new peak at 501 nm confirmed formation of the desired product.

### 4.7 Oligonucleotide Synthesis

The BCN functionalized oligonucleotides **ON1** and **ON2** were synthesized on an ÄKTA oligopilot plus 10 DNA/RNA synthesizer using commercially available building blocks and conventional phosphoramidite strategy. The crude products were purified by reversed‐phase high‐performance liquid chromatography (RP‐HPLC) on a Hypersil ODS C18 column (250 × 4.6 mm, 5 µm) eluting with a linear gradient of MeCN (10—40% over 20 min, flow rate = 1.0 mL min^−1^) in 50 mM aqueous triethylammonium acetate (pH = 7.0). The identity and purity of the products was established by ultra‐performance liquid chromatography electrospray ionization mass spectrometry (UPLC‐ESI‐MS, Figures S4, S5 in the Supporting Information). Concentrations of the stock solutions of pure **ON1** and **ON2** were determined UV spectrophotometrically based on an implementation of the nearest‐neighbors method and assuming molar absorptivity of the BCN moiety to be negligible.

### 4.8 Reaction of Azide Functionalized (6,5)‐SWCNTs With BCN Functionalized Oligonucleotides

An appropriate volume of the aqueous stock solution of either **ON1** or **ON2** (8.4 nmol) was added to a dispersion of **(6,5)‐SWCNT‐N_3_** (*A* = 10 at S_11_) in 1% (*m*/*v*) aqueous SDS (1.0 mL). The mixture was incubated protected from light at 55°C for the 3 d, after which it was filtrated following the procedure described above. The **(6,5)‐SWCNT‐ON1** or **(6,5)‐SWCNT‐ON2** recovered by filtration was resuspended in a 1% (*m*/*v*) aqueous solution of SDS and the resulting dispersion analyzed by UV‐vis‐NIR spectrophotometry. Red‐shift of the main absorption band at around 1000 nm suggested increased polarity around the nanotube, consistent with covalent attachment of the polyanionic oligonucleotide strands.

### 4.9 UV Melting Experiments

UV melting profiles of **(6,5)‐SWCNT‐N_3_**, **(6,5)‐SWCNT‐ON1**, **(6,5)‐SWCNT‐ON2**, and an equimolar mixture of **(6,5)‐SWCNT‐ON1** and **(6,5)‐SWCNT‐ON2** were acquired by a PerkinElmer Lambda 35 spectrophotometer equipped with a Peltier temperature control unit. Appropriate volumes of the stock solutions of the nanotubes were diluted so that in each sample the mass concentration of one type of nanotube was approximately 550 µg L^−1^. The pH of the samples was adjusted to 7.4 with a 20 mM cacodylate buffer and the ionic strength to 0.10 M with NaCl. For homogeneous dispersion, the samples were sonicated vigorously for 3 min and then placed in quartz cuvettes with 10 mm optical path length. Three heating and cooling ramps with a slope of 0.5°C min^−1^ were carried out between 10°C and 90°C, with absorbance at 260 nm recorded at 0.5°C intervals (raw data shown in Figures S5–S8). In the case of the mixture of **(6,5)‐SWCNT‐ON1** and **(6,5)‐SWCNT‐ON2**, sigmoidal hyperchromicity was observed and the melting temperature of this transition was determined as the inflection point of the respective cooling curve.

### 4.10 AFM Experiments

Solutions of the functionalized nanotubes **(6,5)‐SWCNT‐N_3_**, **(6,5)‐SWCNT‐ON1**, **(6,5)‐SWCNT‐ON2** and an equimolar mixture of **(6,5)‐SWCNT‐ON1** and **(6,5)‐SWCNT‐ON2** with a concentration of 550 µg L^−1^, pH of 7.4 (20 mM cacodylate buffer) and ionic strength of 0.10 M (adjusted with NaCl) were sonicated for 10 min in an ultrasonic bath. The silicon wafer substrate cleaning process involved immersion of the wafers in a solution composed of 25% aqueous ammonia, 30% aqueous hydrogen peroxide and deionized water in a volume ratio of 1:1:5, respectively. The solution was heated to approximately 80°C and the wafers were treated for 15 min under gentle stirring. After cleaning, the substrates were thoroughly rinsed with deionized water and dried under a stream of nitrogen gas before sample deposition. 10 µL of the prepared sample solution was drop‐cast onto the silicon wafer surface and allowed to rest for 10 s. The substrate was then spin‐coated at 7200 rpm for 1 min to achieve a uniform surface dispersion. The sample preparation was conducted in a laminar flow cabinet.

Surface characteristics of the prepared samples were characterized using a Park NX‐10 atomic force microscope operated in non‐contact mode. All measurements were performed under ambient conditions using AC160TS cantilevers (Park systems). The acquired AFM images were processed and analyzed using the manufacturer's software (XEI) to extract topographical features.

### 4.11 Photoluminescence Experiments

Steady‐state photoluminescence spectra were measured with an Avantes HS‐TEC spectrometer connected to a 1000 µm diameter optical fiber (Harrick VIS/NIR 0.37NA PC04). The excitation source was an SB522 150 W Xe‐arc lamp coupled to a LOT MSH 300 monochromator. The samples were excited at 570 nm using a wide 50 nm band width.

### 4.12 Statistical Analysis

Defect density of the nanotubes after functionalization with linker **1** was calculated using equation (1). Five Raman spectra were used, giving an arithmetic mean of 150 µm^−1^, with a standard deviation of 10 µm^−1^. In the UV melting profiles presented in Figure 5, each data point is an arithmetic mean from three cooling curves, normalized by division with the absorbance obtained at the low end of the temperature range (10°C). The standard deviation of each data point was less than 1%. These calculations were performed by Microsoft Excel.

## Author Contributions

**Razieh Moosavi**: investigation, writing – original draft. **Tharun K. Kotammagari**: investigation, methodology, writing – review and editing. **Pia Damlin**: data curation, investigation, methodology, writing – review and editing. **Mikko Salomäki**: data curation, investigation, methodology, writing – review and editing. **Mika Lastusaari**: data curation, investigation, methodology, writing – review and editing. **Han Li**: conceptualization, data curation, formal analysis, investigation, methodology, supervision, writing – review and editing. **Tuomas Lönnberg**: conceptualization, data curation, formal analysis, methodology, supervision, visualization, writing – review and editing.

## Conflicts of Interest

The authors declare no conflicts of interest.

## Supporting information




**Supporting File**: chem71205‐sup‐0001‐SuppMat.pdf.

## Data Availability

The data supporting this article have been included as part of the Supporting Information.

## References

[1] J. Ackermann , J. T. Metternich , S. Herbertz , and S. Kruss , “Biosensing With Fluorescent Carbon Nanotubes,” Angewandte Chemie International Edition 61 (2022): e202112372. 10.1002/anie.202112372 PMC9313876 34978752

[2] S. Basu and G. Bisker , “Near‐Infrared Fluorescent Single‐Walled Carbon Nanotubes for Biosensing,” Small 21 (2025): 2502542. 10.1002/smll.202502542 PMC12232249 40317978

[3] M. Zheng , “Sorting Carbon Nanotubes,” Topics in Current Chemistry 375 (2017): 13. 10.1007/s41061-016-0098-z 28083771

[4] M. V. Kharlamova , M. G. Burdanova , M. I. Paukov , and C. Kramberger , “Synthesis, Sorting, and Applications of Single‐Chirality Single‐Walled Carbon Nanotubes,” Materials 15 (2022): 5898. 10.3390/ma15175898 PMC9457432 36079282

[5] T. Feng , Z. Xu , L. Ding , and F. Ding , “Mechanisms of Single‐walled Carbon Nanotube Growth and Progresses on Their Selective Synthesis,” Advanced Functional Materials 35 (2025): e07094.

[6] X. Wei , S. Li , W. Wang , et al., “Recent Advances in Structure Separation of Single‐Wall Carbon Nanotubes and Their Application in Optics, Electronics, and Optoelectronics,” Advancement of Science 9 (2022): 2200054. 10.1002/advs.202200054 PMC9108629 35293698

[7] F. Yang , M. Wang , D. Zhang , J. Yang , M. Zheng , and Y. Li , “Chirality Pure Carbon Nanotubes: Growth, Sorting, and Characterization,” Chemical Reviews 120 (2020): 2693–2758. 10.1021/acs.chemrev.9b00835 32039585

[8] A. S. R. Bati , L. Yu , M. Batmunkh , and J. G. Shapter , “Synthesis, Purification, Properties, and Characterization of Sorted Single‐Walled Carbon Nanotubes,” Nanoscale 10 (2018): 22087–22139. 10.1039/c8nr07379a 30475354

[9] A. Hirsch , “Functionalization of Single‐Walled Carbon Nanotubes,” Angewandte Chemie International Edition 41 (2002): 1853–1859. 10.1002/1521-3773(20020603)41:11<1853::aid-anie1853>3.0.co;2-n 19750614

[10] H. Wang and A. A. Boghossian , “Covalent Conjugation of Proteins Onto Fluorescent Single‐Walled Carbon Nanotubes for Biological and Medical Applications,” Materials Advances 4 (2023): 823–834. 10.1039/d2ma00714b PMC9900427 36761250

[11] X. Yang , X. Zhao , T. Liu , and F. Yang , “Precise Synthesis of Carbon Nanotubes and One‐Dimensional Hybrids From Templates†,” Chinese Journal of Chemistry 39 (2021): 1726–1744.

[12] M. He , D. Wang , Q. Wu , et al., “Designing Catalysts for Chirality‐Selective Synthesis of Single‐Walled Carbon Nanotubes: Past Success and Future Opportunity,” Advanced Materials 31 (2019): 1800805. 10.1002/adma.201800805 30160811

[13] D. Janas , “Towards Monochiral Carbon Nanotubes: a Review of Progress in the Sorting of Single‐walled Carbon Nanotubes,” Materials Chemistry Frontiers 2 (2017): 36–63.

[14] H. Li , C. M. Sims , R. Kang , F. Biedermann , J. A. Fagan , and B. S. Flavel , “Isolation of the (6,5) Single‐wall Carbon Nanotube Enantiomers by Surfactant‐assisted Aqueous Two‐phase Extraction,” Carbon 204 (2023): 475–483.

[15] H. Li , G. Gordeev , O. Garrity , et al., “Separation of Specific Single‐Enantiomer Single‐Wall Carbon Nanotubes in the Large‐Diameter Regime,” ACS Nano 14 (2020): 948–963. 10.1021/acsnano.9b08244 PMC6994058 31742998

[16] H. Li , G. Gordeev , O. Garrity , S. Reich , and B. S. Flavel , “Separation of Small‐diameter Single‐walled Carbon Nanotubes in One to Three Steps With Aqueous Two‐phase Extraction,” ACS Nano 13 (2019): 2567–2578. 10.1021/acsnano.8b09579 30673278

[17] H. Li , M. Zheng , and J. A. Fagan , “Precise Partitioning of Metallic Single‐Wall Carbon Nanotubes and Enantiomers Through Aqueous Two‐Phase Extraction,” ACS Nano 19 (2025): 14137–14149. 10.1021/acsnano.5c00025 PMC12005050 40180889

[18] D. B. Mawhinney , V. Naumenko , A. Kuznetsova , J. T. Yates , J. Liu , and R. E. Smalley , “Surface Defect Site Density on Single Walled Carbon Nanotubes by Titration,” Chemical Physics Letters 324 (2000): 213–216.

[19] M. A. Hamon , H. Hu , P. Bhowmik , et al., “End‐group and Defect Analysis of Soluble Single‐walled Carbon Nanotubes,” Chemical Physics Letters 347 (2001): 8–12.

[20] K. Jiang , A. Eitan , L. S. Schadler , et al., “Selective Attachment of Gold Nanoparticles to Nitrogen‐doped Carbon Nanotubes,” Nano Letters 3 (2003): 275–277.

[21] J. Chen , M. A. Hamon , H. Hu , et al., “Solution Properties of Single‐walled Carbon Nanotubes,” Science 282 (1998): 95–98. 10.1126/science.282.5386.95 9756485

[22] R. Yu , L. Chen , Q. Liu , et al., “Platinum Deposition on Carbon Nanotubes via Chemical Modification,” Chemistry of Materials 10 (1998): 718–722.

[23] M. Holzinger , O. Vostrowsky , A. Hirsch , et al., “Sidewall Functionalization of Carbon Nanotubes,” Angewandte Chemie International Edition 40 (2001): 4002–4005. 10.1002/1521-3773(20011105)40:21<4002::AID-ANIE4002>3.0.CO;2-8 29712262

[24] V. Georgakilas , K. Kordatos , M. Prato , D. M. Guldi , M. Holzinger , and A. Hirsch , “Organic Functionalization of Carbon Nanotubes,” Journal of the American Chemical Society 124 (2002): 760–761. 10.1021/ja016954m 11817945

[25] K. Kamaras , M. E. Itkis , H. Hu , B. Zhao , and R. C. Haddon , “Covalent Bond Formation to a Carbon Nanotube Metal,” Science 301 (2003): 1501. 10.1126/science.1088083 12970557

[26] M. Zheng , A. Jagota , E. D. Semke , et al., “DNA‐assisted Dispersion and Separation of Carbon Nanotubes,” Nature Materials 2 (2003): 338–342. 10.1038/nmat877 12692536

[27] M. Zheng , A. Jagota , M. S. Strano , et al., “Structure‐Based Carbon Nanotube Sorting by Sequence‐Dependent DNA Assembly,” Science 302 (2003): 1545–1548. 10.1126/science.1091911 14645843

[28] Y. Zheng , S. M. Bachilo , and R. B. Weisman , “Controlled Patterning of Carbon Nanotube Energy Levels by Covalent DNA Functionalization,” ACS Nano 13 (2019): 8222–8228. 10.1021/acsnano.9b03488 31244048

[29] Z. Lin , L. C. Beltran , Z. A. De los Santos , et al., “DNA‐guided Lattice Remodeling of Carbon Nanotubes,” Science 377 (2022): 535–539. 10.1126/science.abo4628 PMC9872717 35901135

[30] P. Galonska , J. M. Mohr , C. A. Schrage , L. Schnitzler , and S. Kruss , “Guanine Quantum Defects in Carbon Nanotubes for Biosensing,” Journal of Physical Chemistry Letters 14 (2023): 3483–3490. 10.1021/acs.jpclett.3c00358 37011259

[31] G. Amoroso , Q. Ye , K. Cervantes‐Salguero , G. Fernández , A. Cecconello , and M. Palma , “DNA‐Powered Stimuli‐Responsive Single‐Walled Carbon Nanotube Junctions,” Chemistry of Materials 31 (2019): 1537–1542.

[32] Z. Mengrani , W. Hong , and M. Palma , “DNA‐Mediated Carbon Nanotubes Heterojunction Assembly,” ACS Nanoscience Au 4 (2024): 391–398. 10.1021/acsnanoscienceau.4c00025 PMC11659895 39713723

[33] M. Freeley , R. E. A. Gwyther , D. D. Jones , and M. Palma , “DNA‐directed Assembly of Carbon Nanotube–protein Hybrids,” Biomolecules 11 (2021): 955. 10.3390/biom11070955 PMC8301810 34209628

[34] N. J. Agard , J. A. Prescher , and C. R. Bertozzi , “A Strain‐promoted [3 + 2] Azide‐alkyne Cycloaddition for Covalent Modification of Biomolecules in Living Systems,” Journal of the American Chemical Society 126 (2004): 15046–15047. 10.1021/ja044996f 15547999

[35] G. Manoharan , P. Bösel , J. Thien , et al., “Click‐Functionalization of Silanized Carbon Nanotubes: From Inorganic Heterostructures to Biosensing Nanohybrids,” Molecules 28 (2023): 2161. 10.3390/molecules28052161 PMC10004328 36903408

[36] N. F. Hartmann , K. A. Velizhanin , E. H. Haroz , et al., “Photoluminescence Dynamics of Aryl sp^3^ Defect States in Single‐Walled Carbon Nanotubes,” ACS Nano 10 (2016): 8355–8365. 10.1021/acsnano.6b02986 27529740

[37] F. J. Berger , J. Lüttgens , T. Nowack , et al., “Brightening of Long, Polymer‐Wrapped Carbon Nanotubes by sp^3^ Functionalization in Organic Solvents,” ACS Nano 13 (2019): 9259–9269. 10.1021/acsnano.9b03792 PMC6716210 31381849

[38] S. S. Piletsky , E. E. Keblish , and D. A. Heller , “Controlled Quantum Well Formation on DNA‐Wrapped Carbon Nanotubes via Peroxide‐Mediated Aryl Diazonium Reduction,” Nano Letters 25 (2025): 2480–2485. 10.1021/acs.nanolett.4c06061 PMC12191770 39898587

[39] F. A. Mann , N. Herrmann , F. Opazo , and S. Kruss , “Quantum Defects as a Toolbox for the Covalent Functionalization of Carbon Nanotubes With Peptides and Proteins,” Angewandte Chemie International Edition 59 (2020): 17732–17738. 10.1002/anie.202003825 PMC7540668 32511874

[40] J. L. Bahr , J. Yang , D. V. Kosynkin , M. J. Bronikowski , R. E. Smalley , and J. M. Tour , “Functionalization of Carbon Nanotubes by Electrochemical Reduction of Aryl Diazonium Salts: a Bucky Paper Electrode,” Journal of the American Chemical Society 123 (2001): 6536–6542. 10.1021/ja010462s 11439040

[41] G. Schmidt , S. Gallon , S. Esnouf , J. P. Bourgoin , and P. Chenevier , “Mechanism of the Coupling of Diazonium to Single‐Walled Carbon Nanotubes and Its Consequences,” Chemistry: A European Journal 15 (2009): 2101–2110. 10.1002/chem.200801801 19142944

[42] M. Gomberg and W. E. Bachmann , “The Synthesis of Biaryl Compounds by Means of the Diazo Reaction,” Journal of the American Chemical Society 46 (1924): 2339–2343.

[43] T. Wei , J. C. Furgal , and T. F. Scott , “In Situ Deprotection and Dynamic Covalent Assembly Using a Dual Role Catalyst,” Chemical Communications 53 (2017): 3874–3877. 10.1039/c7cc01028a 28317995

[44] F. L. Sebastian , F. Becker , Y. Yomogida , et al., “Unified Quantification of Quantum Defects in Small‐Diameter Single‐Walled Carbon Nanotubes by Raman Spectroscopy,” ACS Nano 17 (2023): 21771–21781. 10.1021/acsnano.3c07668 PMC10655237 37856164

[45] P. Wang , J. Fortner , H. Luo , et al., “Quantum Defects: What Pairs With the Aryl Group When Bonding to the sp^2^ Carbon Lattice of Single‐Wall Carbon Nanotubes?,” Journal of the American Chemical Society 144 (2022): 13241. 10.1021/jacs.2c03846 35830302

[46] G. P. Evans , D. J. Buckley , N. T. Skipper , and I. P. Parkin , “Switchable Changes in the Conductance of Single‐walled Carbon Nanotube Networks on Exposure to Water Vapour,” Nanoscale 9 (2017): 11279–11287. 10.1039/c7nr02141k 28758671

[47] C. Valleroy , R. d'Ambrosio , C. Blanc , E. Anglaret , L. Firlej , and C. Wexler , “Temperature Dependence of the Near Infrared Absorption Spectrum of Single‐wall Carbon Nanotubes Dispersed by Sodium Dodecyl Sulfate in Aqueous Solution: Experiments and Molecular Dynamics Study,” Journal of Molecular Modeling 30 (2024): 1–11. 10.1007/s00894-024-06068-y 39066924

[48] M. Abdolkarimi‐Mahabadi , A. Bayat , and A. Mohammadi , “Use of UV‐Vis Spectrophotometry for Characterization of Carbon Nanostructures: A Review,” Theoretical and Experimental Chemistry 57 (2021): 191–198.

[49] B. J. Gifford , S. Kilina , H. Htoon , S. K. Doorn , and S. Tretiak , “Exciton Localization and Optical Emission in Aryl‐Functionalized Carbon Nanotubes,” Journal of Physical Chemistry C 122 (2018): 1838.

[50] Y. Zheng , Y. Han , B. M. Weight , et al., “Photochemical Spin‐state Control of Binding Configuration for Tailoring Organic Color Center Emission in Carbon Nanotubes,” Nature Communications 13 (2022): 4439. 10.1038/s41467-022-31921-0 PMC9343348 35915090

[51] F. L. Sebastian , S. Settele , H. Li , B. S. Flavel , and J. Zaumseil , “How to Recognize Clustering of Luminescent Defects in Single‐wall Carbon Nanotubes,” Nanoscale Horizons 9 (2024): 2294. 10.1039/d4nh00383g PMC11462117 39380328

[52] M. E. Hughes , E. Brandin , and J. A. Golovchenko , “Optical Absorption of DNA‐carbon Nanotube Structures,” Nano Letters 7 (2007): 1191–1194. 10.1021/nl062906u PMC3183501 17419658

[53] H. Huang , H. Kajiura , R. Maruyama , K. Kadono , and K. Noda , “Relative Optical Absorption of Metallic and Semiconducting Single‐walled Carbon Nanotubes,” Journal of Physical Chemistry B 110 (2006): 4686–4690. 10.1021/jp054839i 16526703

[54] Y. Zheng , S. R. Sanchez , S. M. Bachilo , and R. B. Weisman , “Indexing the Quality of Single‐Wall Carbon Nanotube Dispersions Using Absorption Spectra,” Journal of Physical Chemistry C 122 (2018): 4681–4690.

[55] Y. Li , X. Han , and Z. Deng , “Grafting Single‐Walled Carbon Nanotubes With Highly Hybridizable DNA Sequences: Potential Building Blocks for DNA‐Programmed Material Assembly,” Angewandte Chemie International Edition 46 (2007): 7481–7484. 10.1002/anie.200701748 17691092

[56] S. K. Maity and T. Lönnberg , “Oligonucleotides Incorporating Palladacyclic Nucleobase Surrogates,” Chemistry: A European Journal 24 (2018): 1274–1277. 10.1002/chem.201705797 29227000

