# Supplementary material for: Successive Azide and DNA Functionalization for Reversible and Controlled Association of Single‐Wall Carbon Nanotubes
Source: Chemistry. 2026 Jun 1;32(35):e71205. doi: 10.1002/chem.71205 (PMC13587638; doi:10.1002/chem.71205)
Supplement: Supplementary file 1 [file CHEM-32-e71205-s001.pdf]

Supporting Information

**Successive Azide and DNA Functionalization for Reversible and Controlled Association of Single-Wall Carbon Nanotubes**

*Razieh Moosavi, Tharun K. Kotammagari, Pia Damlin, Mikko Salomäki, Mika Lastusaari, Han Li* and Tuomas Lönnberg**

**Contents**

**Figure S1.** A figure highlighting the high volume, concentration and purity of the (6,5)-SWCNT starting material. S2
**Figure S2.** $^{1}$H NMR spectrum of compound **1** (500 MHz, $D_2O/CD_3OD$). S3
**Figure S3.** $^{13}$C NMR spectrum of compound **1** (126 MHz, $D_2O/CD_3OD$). S5
**Figure S4.** A) UV and extracted ion UPLC traces and B) mass spectrum of oligonucleotide **ON1**. S6
**Figure S5.** A) UV and extracted ion UPLC traces and B) mass spectrum of oligonucleotide **ON2**. S7
**Figure S6.** UV absorbance of **(6,5)-SWCNT-N$_3$** as a function of temperature. S8
**Figure S7.** UV absorbance of **(6,5)-SWCNT-ON1** as a function of temperature. S9
**Figure S8.** UV absorbance of **(6,5)-SWCNT-ON2** as a function of temperature. S10
**Figure S9.** UV absorbance of a mixture of **(6,5)-SWCNT-ON1** and **(6,5)-SWCNT-ON2** as a function of temperature. S11
**Figure S10.** Samples of the UV melting experiments after three heating and cooling cycles. S12
**Figure S11.** Height profile analysis of **(6,5)-SWCNT-N$_3$**. S12
**Figure S12.** Height profile analysis of **(6,5)-SWCNT-ON1**. S13
**Figure S13.** Height profile analysis of **(6,5)-SWCNT-ON2**. S13
**Figure S14.** Height profile analysis of a mixture of **(6,5)-SWCNT-ON1** and **(6,5)-SWCNT-ON2**. S14
**Figure S15.** Representative Raman spectra of two batches of **(6,5)-SWCNT-N$_3$**. S14
**Figure S16.** Emission spectra of pristine **(6,5)-SWCNT** and **(6,5)-SWCNT-N$_3$**. S15

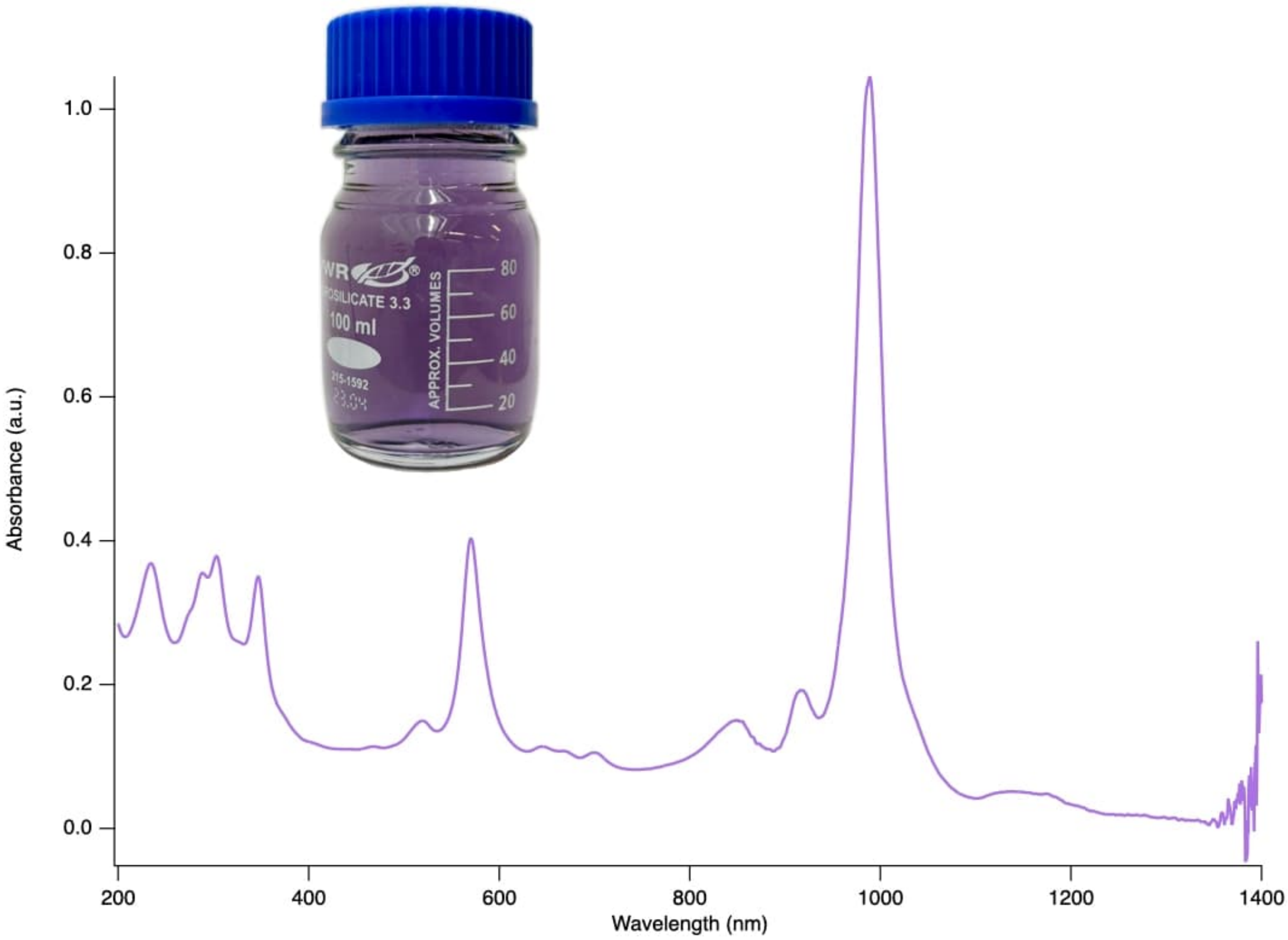


**Figure S1.** A figure highlighting the high volume, concentration and purity of the (6,5)-SWCNT starting material.

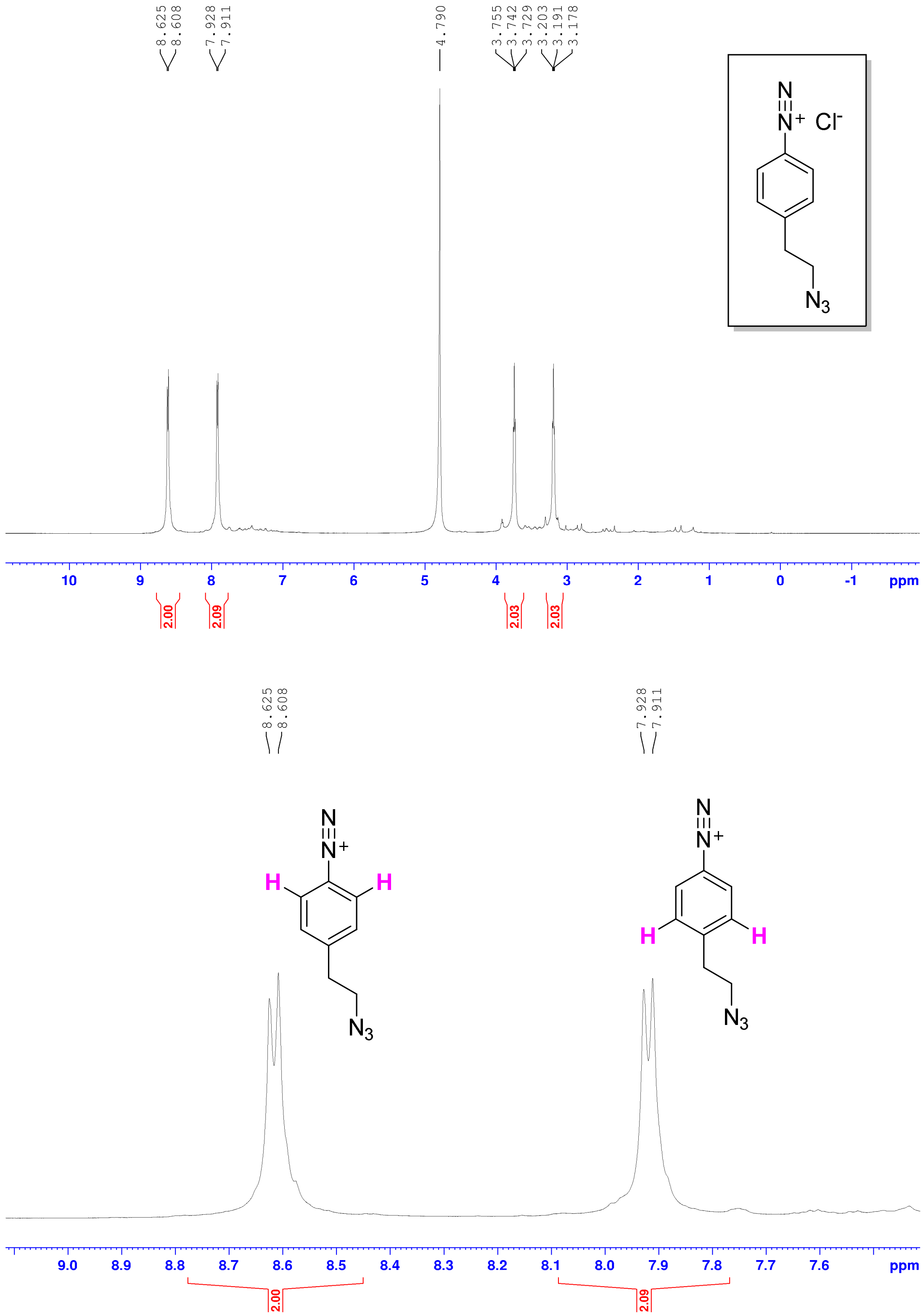


**Figure S2.** $^{1}H$ NMR spectrum of compound **1** (500 MHz, $D_2O/CD_3OD$).

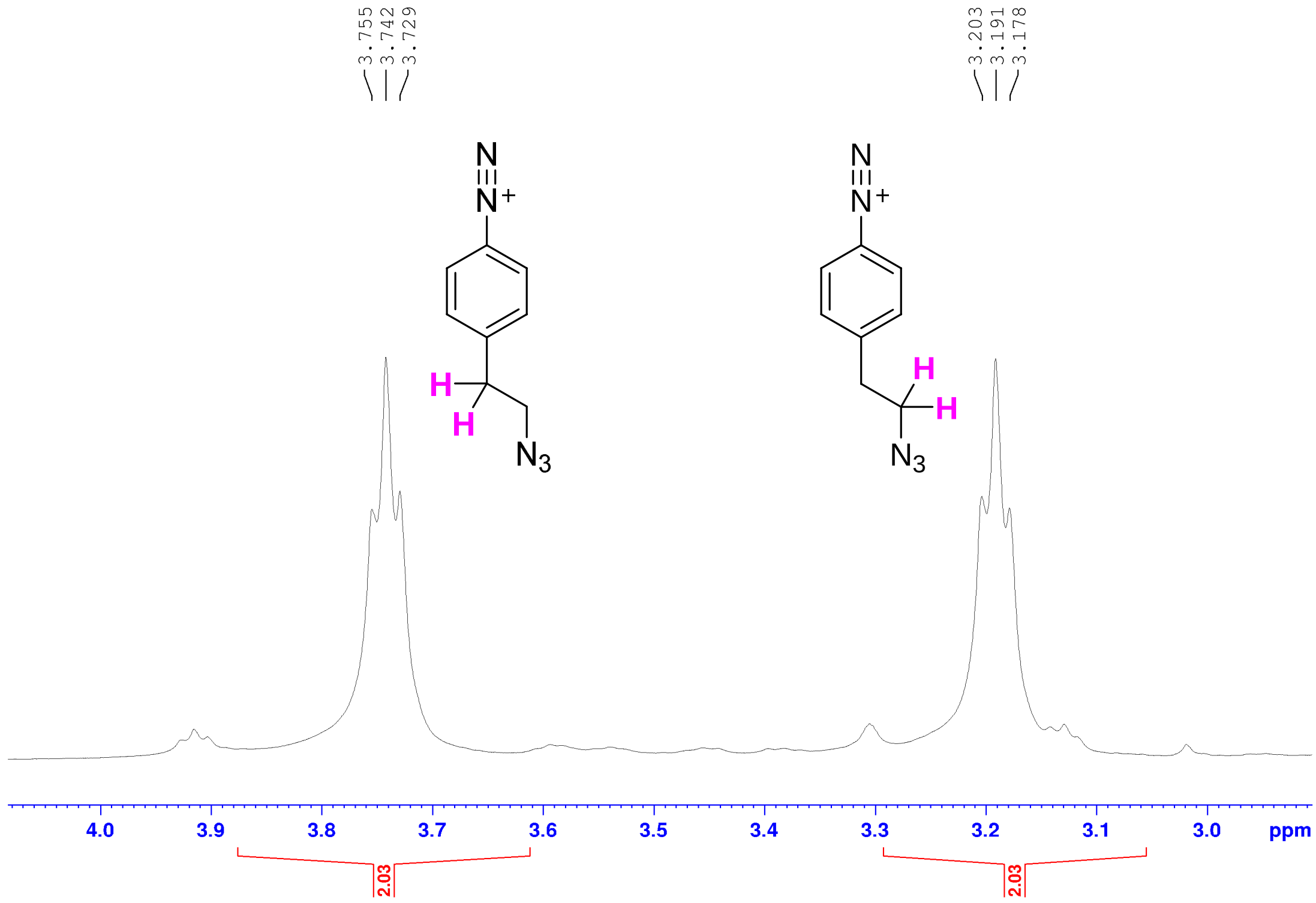


**Figure S2 (continued).** $^{1}$H NMR spectrum of compound **1** (500 MHz, $D_2O/CD_3OD$).

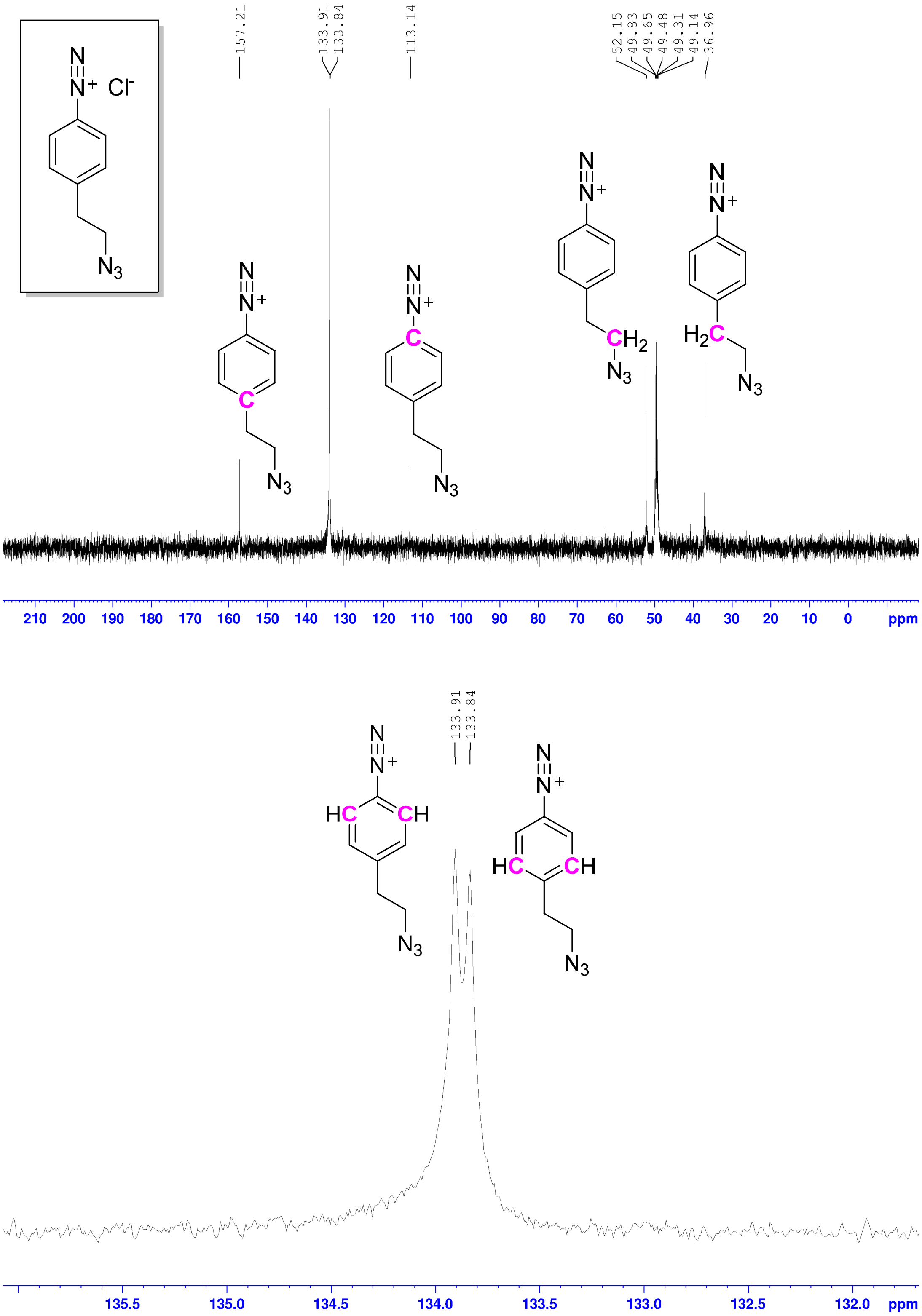


**Figure S3.** $^{13}$C NMR spectrum of compound **1** (126 MHz, $D_2O/CD_3OD$).

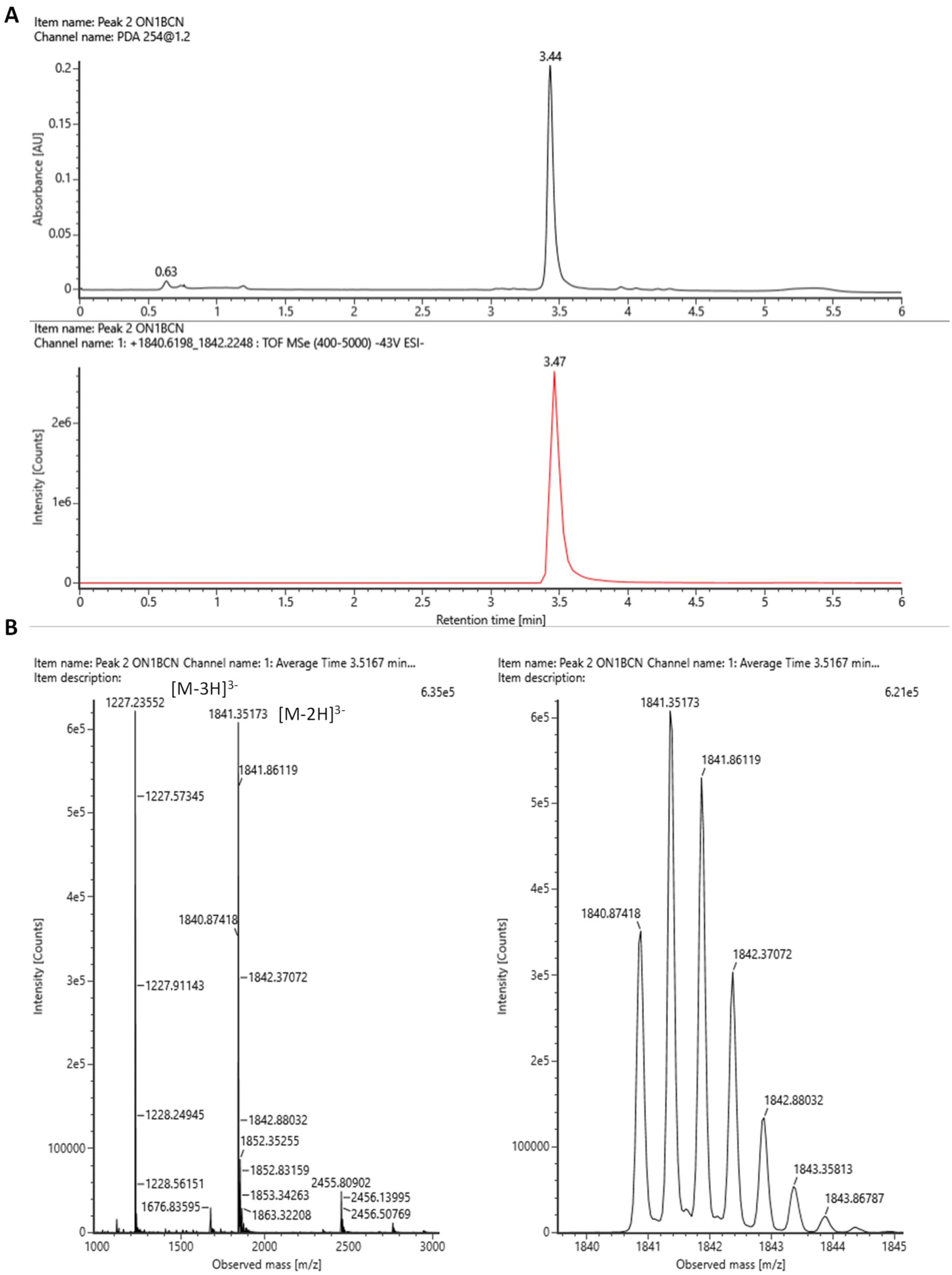


**Figure S4.** A) UV and extracted ion UPLC traces and B) mass spectrum of oligonucleotide **ON1**; ACQUITY Premier OST column (50 × 2.1 mm, 1.7 µm); flow rate 0.4 mL min$^{-1}$; linear gradient (5—25 % over 4 min) of MeOH in aqueous solution of hexafluoroisopropanol (40 mM) and triethylamine (7 mM); $\lambda$ = 254 nm; $T$ = 60 °C.

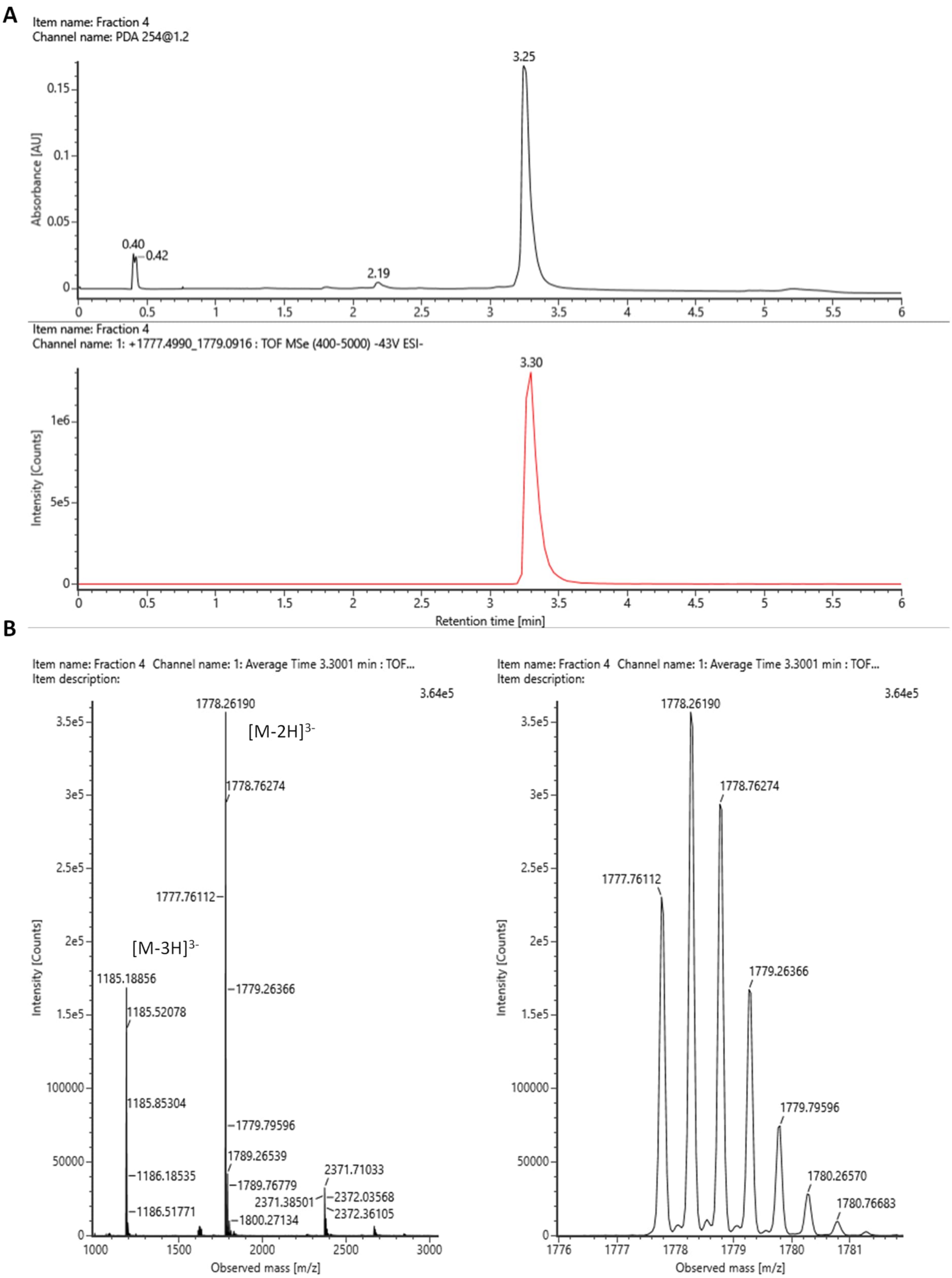


**Figure S5.** A) UV and extracted ion UPLC traces and B) mass spectrum of oligonucleotide **ON2**; ACQUITY Premier OST column (50 × 2.1 mm, 1.7 µm); flow rate 0.4 mL $min^{-1}$; linear gradient (5—25 % over 4 min) of MeOH in aqueous solution of hexafluoroisopropanol (40 mM) and triethylamine (7 mM); $\lambda$ = 254 nm; $T$ = 60 °C.

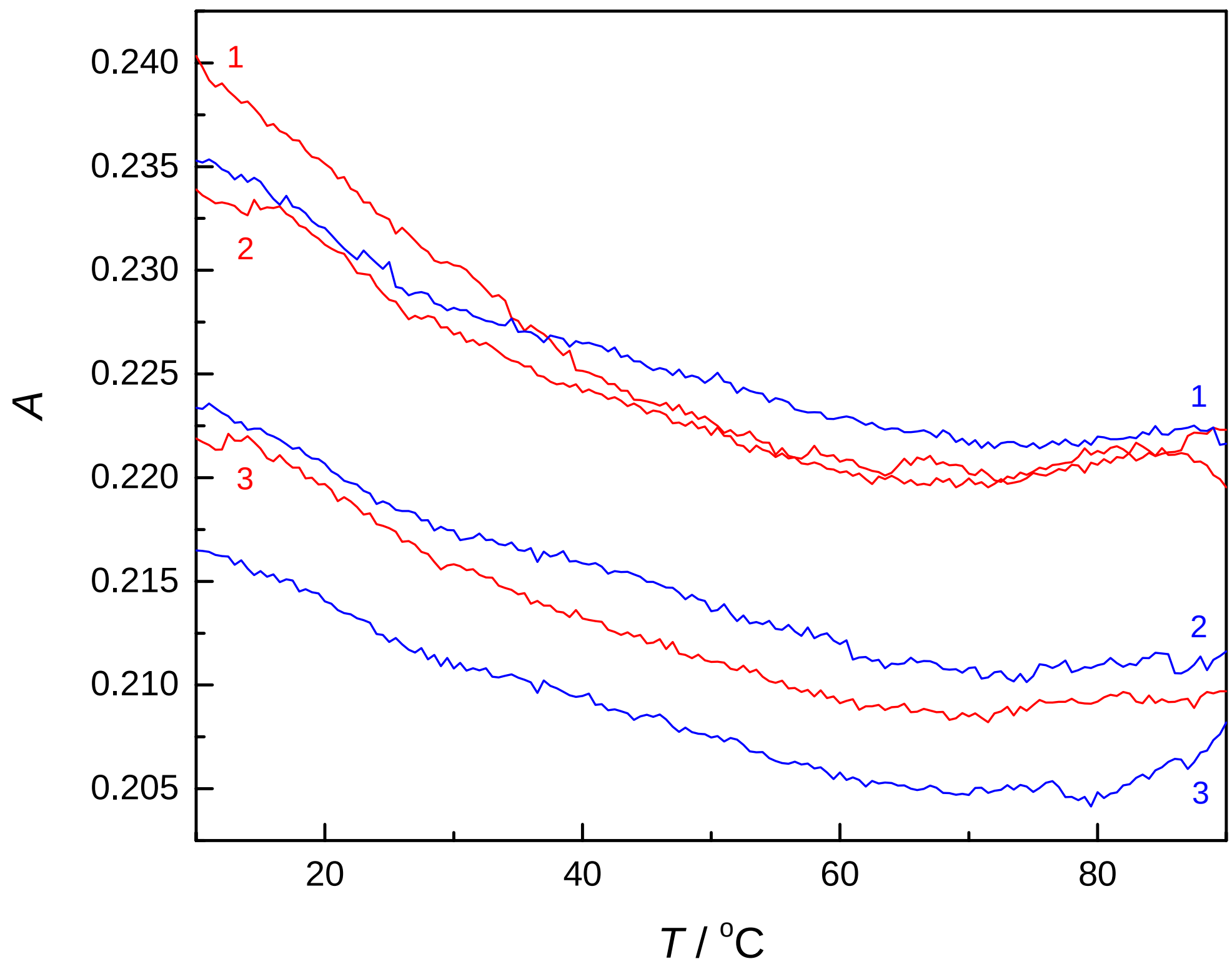


**Figure S6.** UV absorbance of **(6,5)-SWCNT-N3** as a function of temperature; $\rho$(nanotubes) = 550 µg $L^{-1}$; pH = 7.4 (20 mM cacodylate buffer); *I*(NaCl) = 0.10 M; 1 % (*m*/*v*) SDS. The red lines refer to heating and the blue lines to cooling ramps and the numbers (1—3) indicate the cycle.

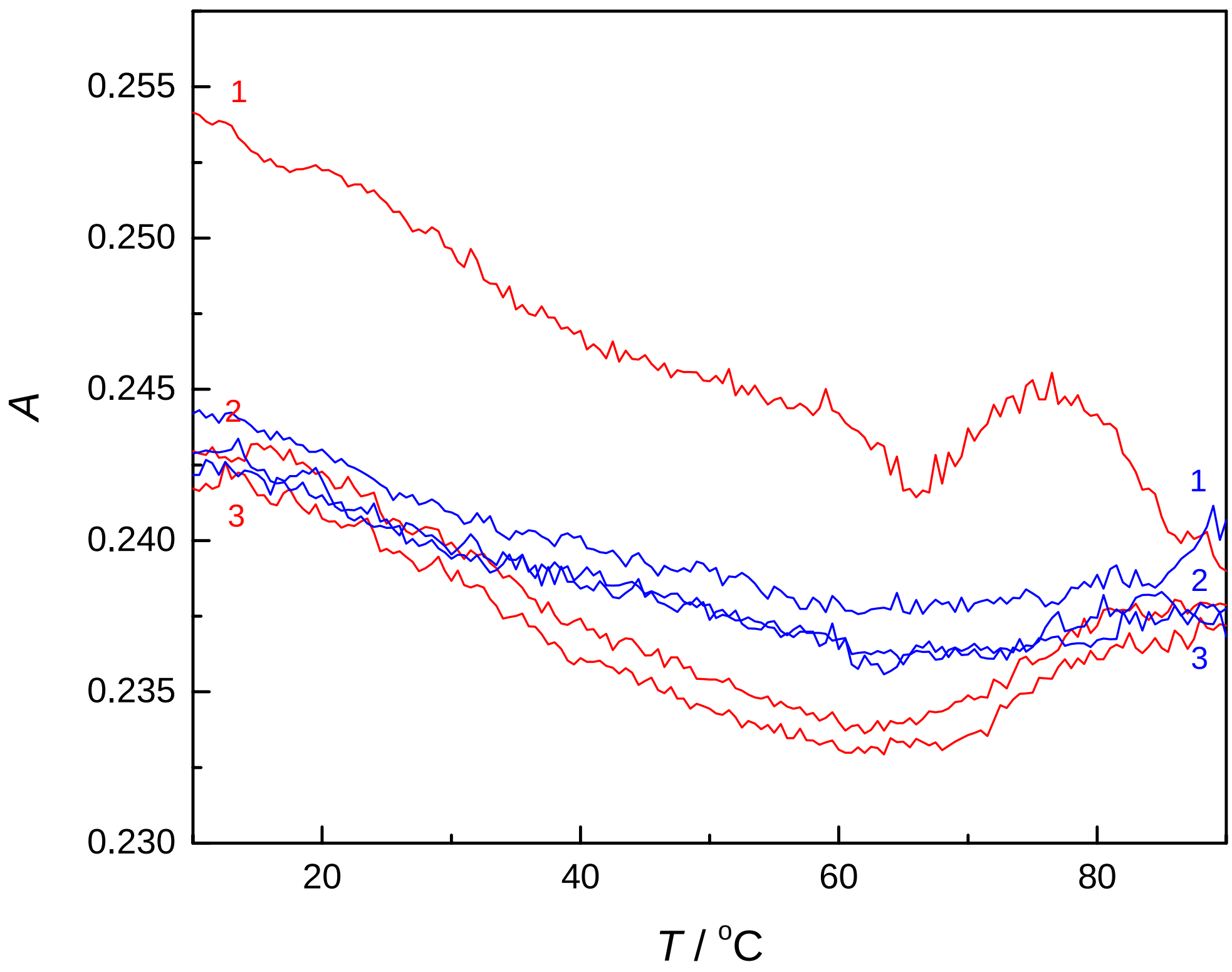


**Figure S7.** UV absorbance of **(6,5)-SWCNT-ON1** as a function of temperature; $\rho$(nanotubes) = 550 µg $L^{-1}$; pH = 7.4 (20 mM cacodylate buffer); $I$(NaCl) = 0.10 M; 1 % ($m$/$v$) SDS. The red lines refer to heating and the blue lines to cooling ramps and the numbers (1—3) indicate the cycle.

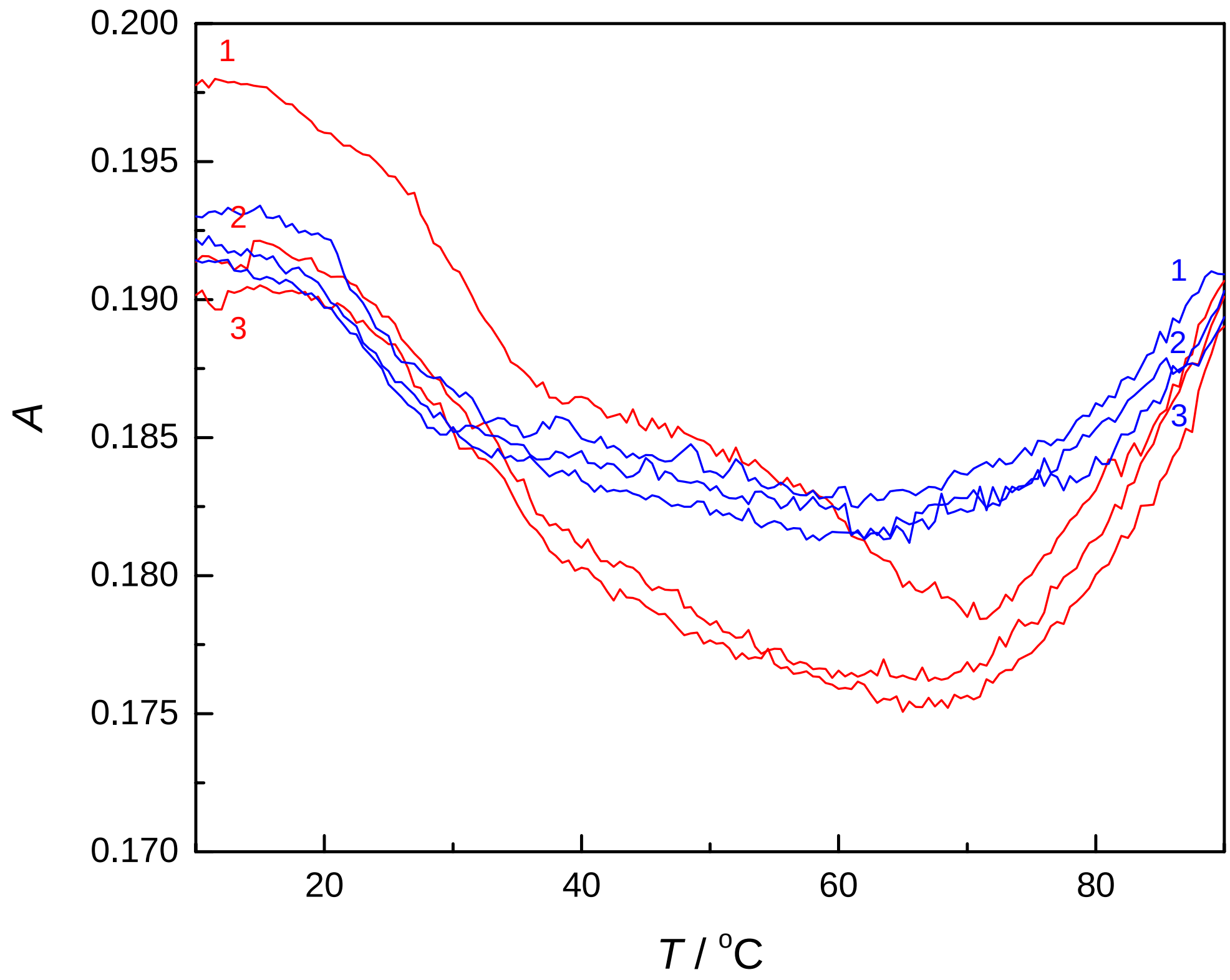


**Figure S8.** UV absorbance of **(6,5)-SWCNT-ON2** as a function of temperature; $\rho$(nanotubes) = 550 μg L$^{-1}$; pH = 7.4 (20 mM cacodylate buffer); *I*(NaCl) = 0.10 M; 1 % (*m*/*v*) SDS. The red lines refer to heating and the blue lines to cooling ramps and the numbers (1—3) indicate the cycle.

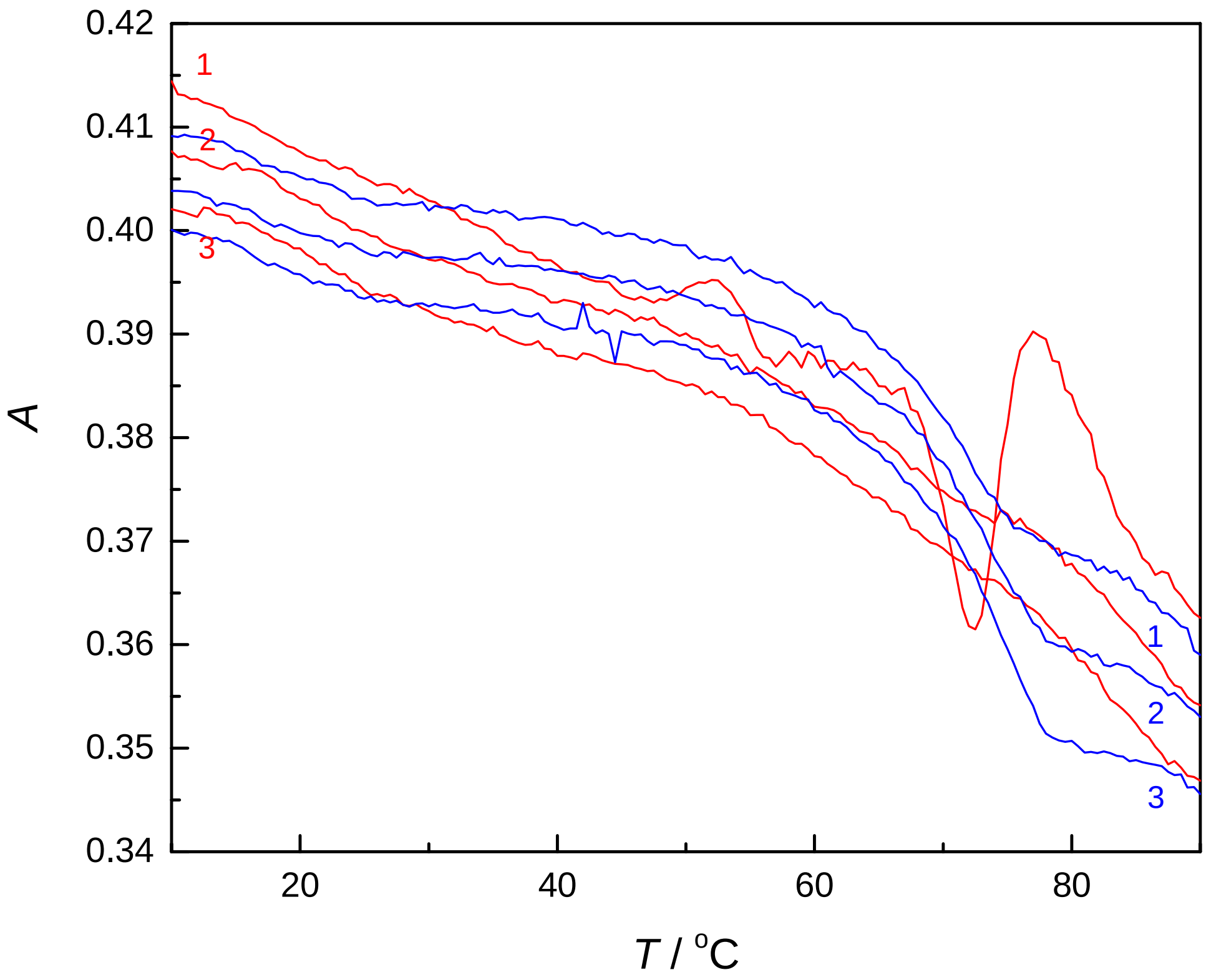


**Figure S9.** UV absorbance of a mixture of **(6,5)-SWCNT-ON1** and **(6,5)-SWCNT-ON2** as a function of temperature; $\rho$(nanotubes) = 550 µg $L^{-1}$; pH = 7.4 (20 mM cacodylate buffer); $I$(NaCl) = 0.10 M; 1 % (*m*/*v*) SDS. The red lines refer to heating and the blue lines to cooling ramps and the numbers (1—3) indicate the cycle.

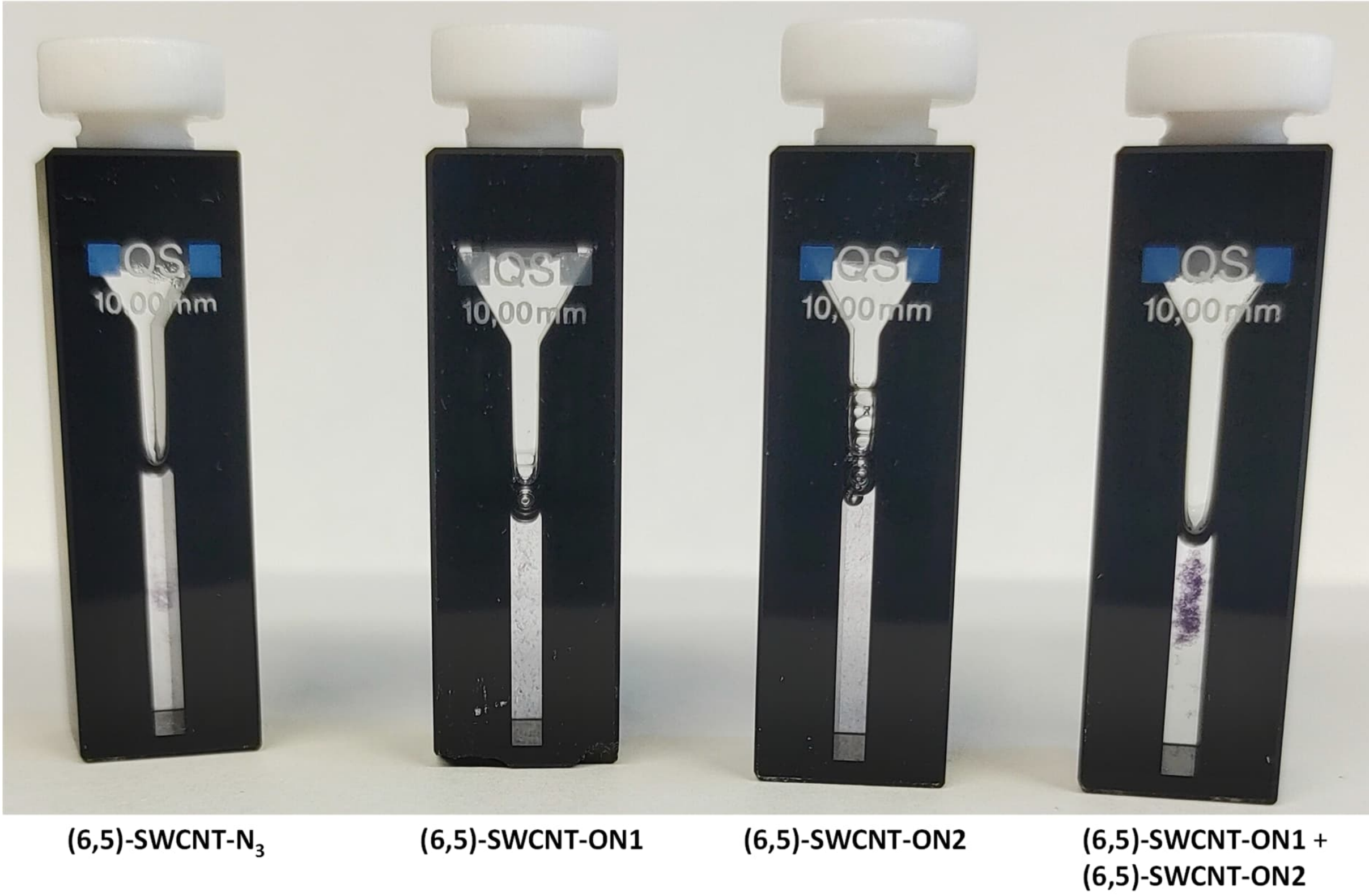


**Figure S10.** Samples of the UV melting experiments after three heating and cooling cycles.

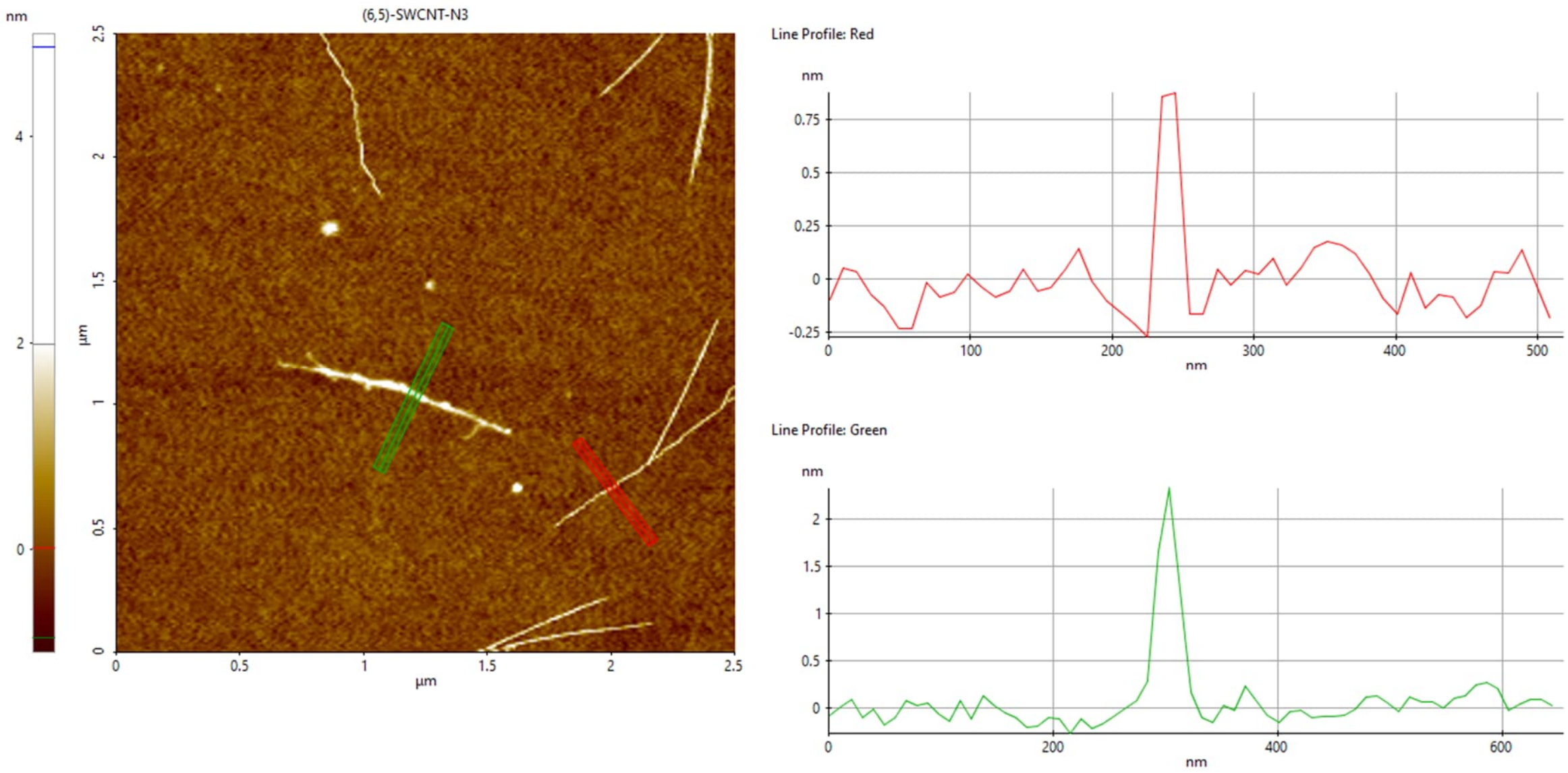


**Figure S11.** Height profile analysis of **(6,5)-SWCNT-$N_3$** obtained from AFM topography image.

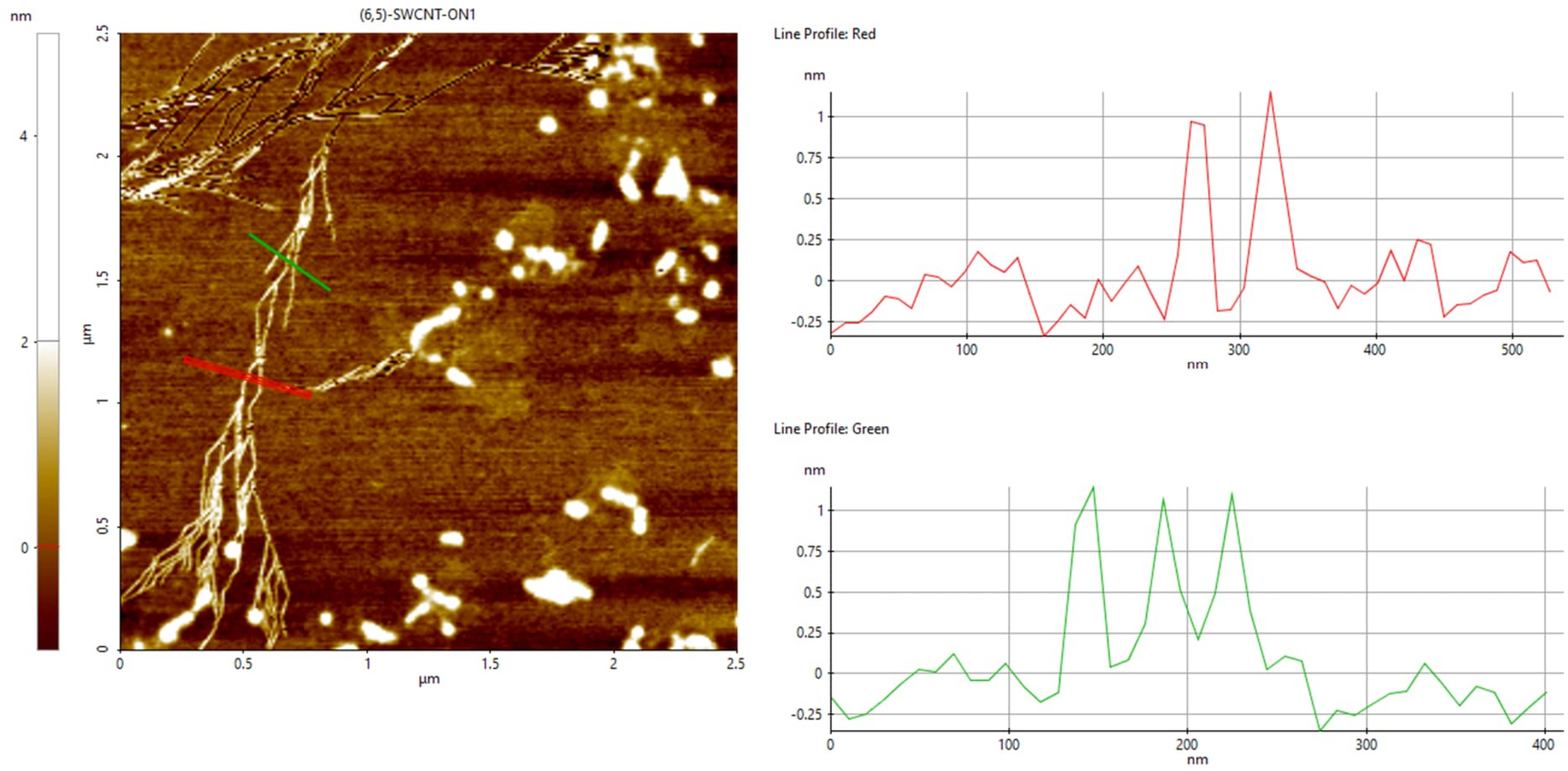


**Figure S12.** Height profile analysis of **(6,5)-SWCNT-ON1** obtained from AFM topography image.

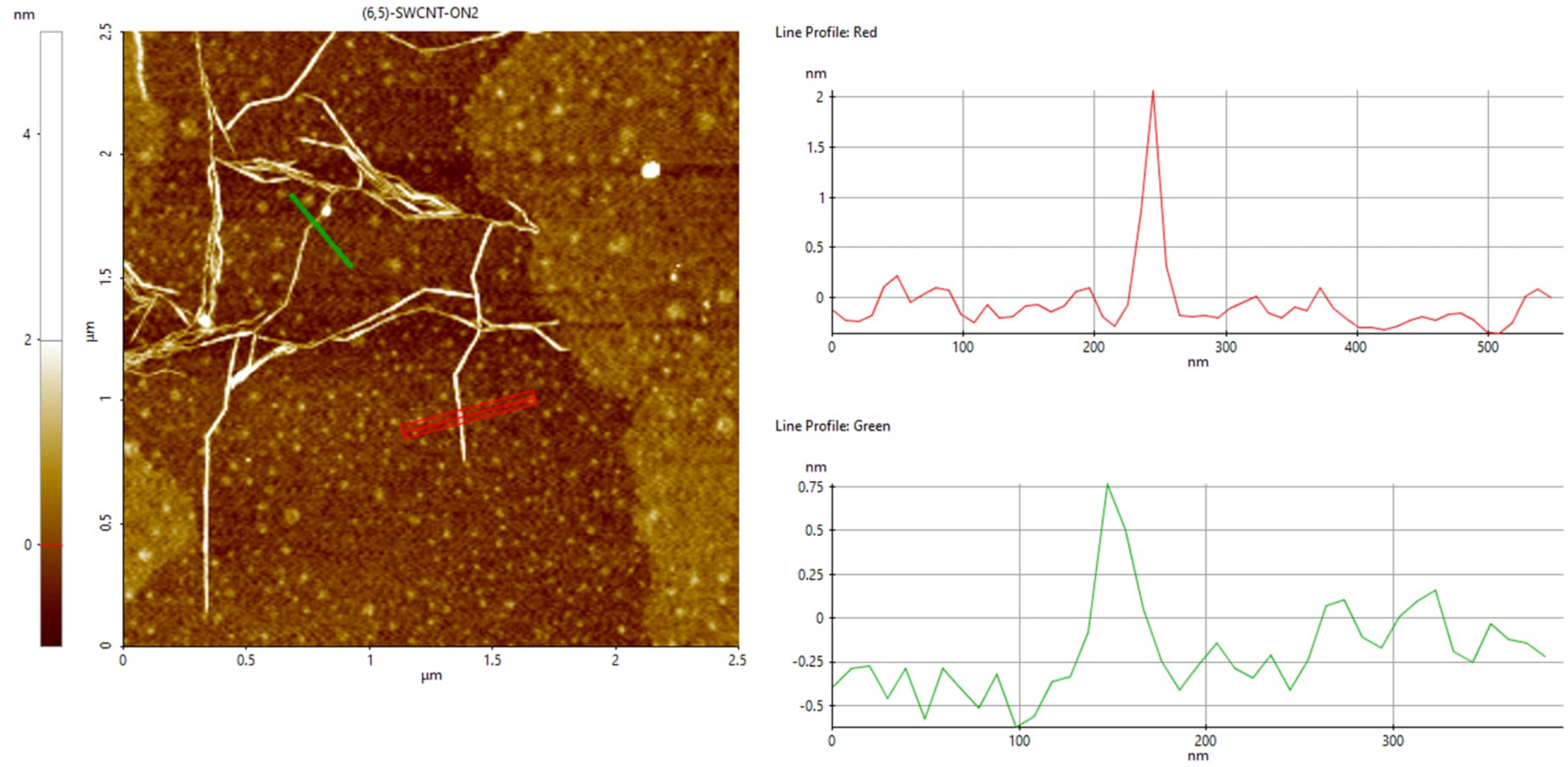


**Figure S13.** Height profile analysis of **(6,5)-SWCNT-ON2** obtained from AFM topography image.

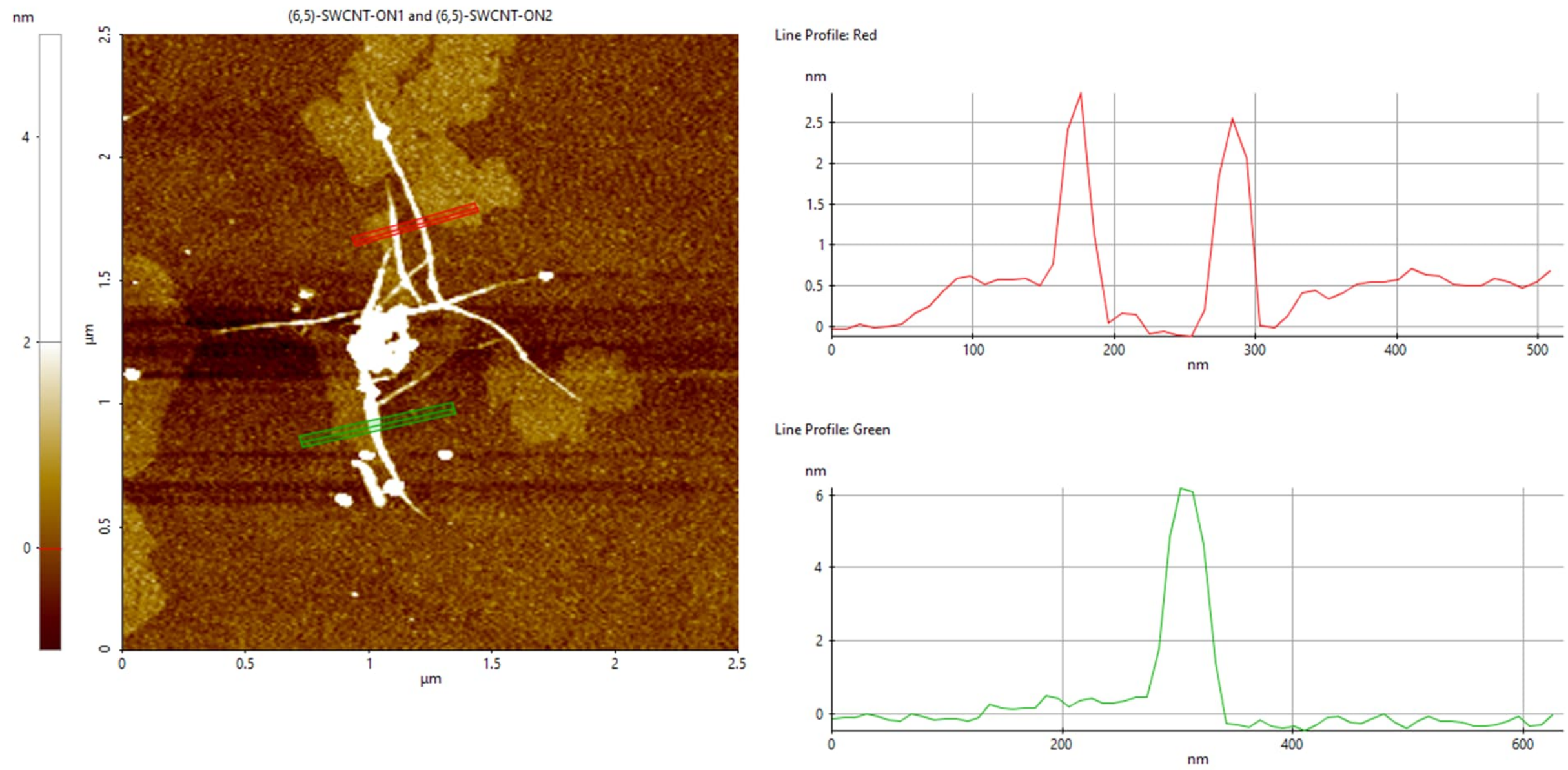


**Figure S14.** Height profile analysis of a mixture of **(6,5)-SWCNT-ON1** and **(6,5)-SWCNT-ON2** obtained from AFM topography image.

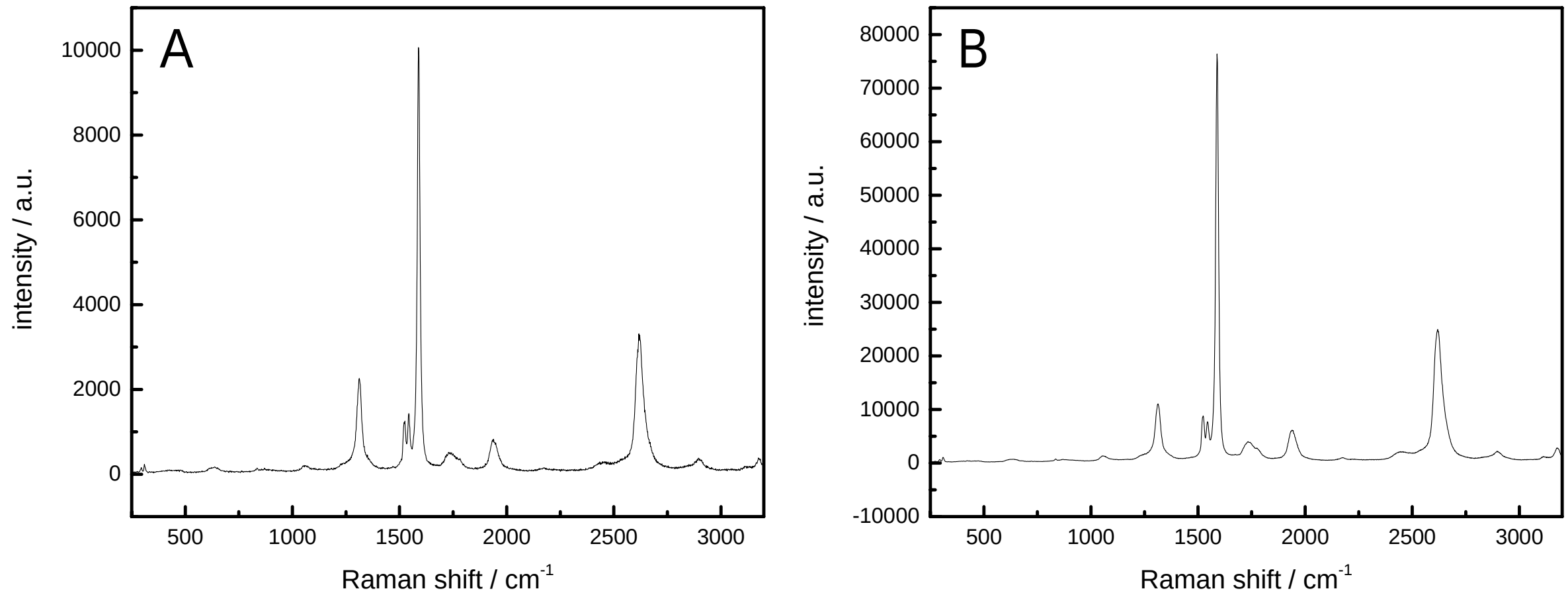


**Figure S15.** Representative Raman spectra of two batches of **(6,5)-SWCNT-N$_3$**. Spectrum A is from the material used in all of the other experiments and spectrum B from a smaller (6 %) batch prepared to test the reproducibility of the azide functionalization. Defect densities of these batches were 150 and 110 µm$^{-1}$, respectively.

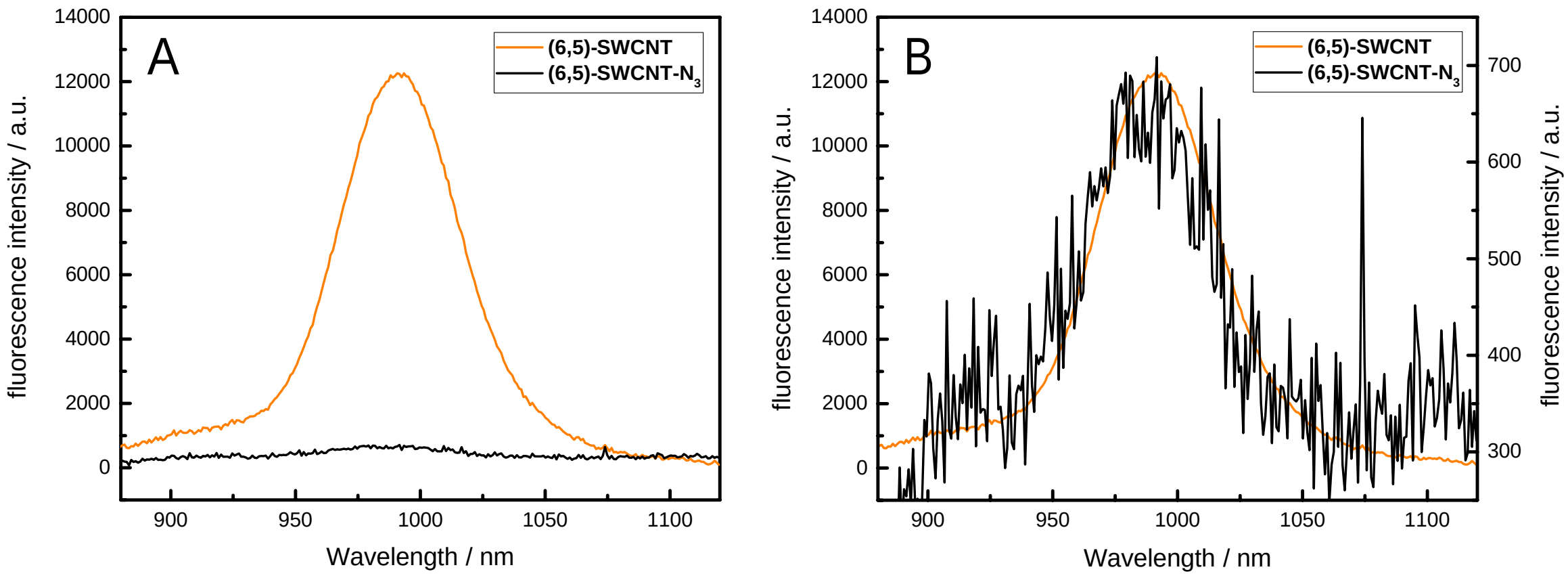


**Figure S16.** Emission spectra of pristine **(6,5)-SWCNT** (orange line) and **(6,5)-SWCNT-$N_3$** (black line). The spectra were normalized to absorption of the samples at the excitation wavelength (570 nm). Plot A shows the two spectra in scale, while the double-y-axis plot B has been included to illustrate the fact that while the $E_{11}$ emission peak (at 981 nm) is greatly diminished on functionalization, it still retains the characteristic shape and position.
